# Functional Multigenomic Screening of Human-Associated Bacteria for NF-κB-Inducing Bioactive Effectors

**DOI:** 10.1128/mBio.02587-19

**Published:** 2019-11-19

**Authors:** Andreia B. Estrela, Toshiki G. Nakashige, Christophe Lemetre, Ian D. Woodworth, Jazz L. Weisman, Louis J. Cohen, Sean F. Brady

**Affiliations:** a Laboratory of Genetically Encoded Small Molecules, The Rockefeller University, New York, New York, USA; b Division of Gastroenterology, Icahn School of Medicine at Mount Sinai, New York, New York, USA; Hudson Institute of Medical Research; Nanyang Technological University

**Keywords:** NF-κB, functional screening, human microbiome

## Abstract

The effect of the microbiota on its human host is driven, at least in part, by small-molecule and protein effectors it produces. Here, we report on the use of functional multigenomic screening to identify microbiota-encoded effectors. In this study, genomic DNA from 116 human-associated bacteria was cloned *en masse*, and the resulting multigenomic library was screened using a nuclear factor-κB reporter (NF-κB) assay. Functional multigenomics builds on the concept of functional metagenomics but takes advantage of increasing advances in cultivating and sequencing human-associated bacteria. Effector genes found to confer NF-κB-inducing activity to Escherichia coli encode proteins in four general categories: cell wall hydrolases, membrane transporters, lipopolysaccharide biosynthetic enzymes, and proteins of unknown function. The compact nature of multigenomic libraries, which results from the ability to normalize input DNA ratios, should simplify screening of libraries using diverse heterologous hosts and reporter assays, increasing the rate of discovery of novel effector genes.

## Abstract (precis)

Human-associated bacteria are thought to encode bioactive small molecules and proteins that play an intimate role in human health and disease. Here, we report on the creation and functional screening of a multigenomic library constructed using genomic DNA from 116 bacteria found at diverse sites across the human body. Individual clones were screened for genes capable of conferring NF-κB-inducing activity to Escherichia coli. NF-κB is a useful reporter for a range of cellular processes related to immunity, pathogenesis, and inflammation. Compared to the screening of metagenomic libraries, the ability to normalize input DNA ratios when constructing a multigenomic library should facilitate the more efficient examination of commensal bacteria for diverse bioactivities. Multigenomic screening takes advantage of the growing available resources in culturing and sequencing the human microbiota and generates starting points for more in-depth studies on the mechanisms by which commensal bacteria interact with their human host.

## INTRODUCTION

A diverse and dynamic community of microorganisms inhabits the human body. Although these microbes are thought to play an important role in human health and disease, the mechanisms by which they interact with their human host are still largely a mystery (1). One method that has been explored for identifying bacterial small molecules and proteins that affect human physiology (i.e., effectors) is functional metagenomics (2–4). In these studies, DNA is extracted directly from a host microbial community, most commonly stool, and cloned into model systems where the resulting libraries are then screened for bioactivities in high-throughput phenotypic assays. Functional metagenomics was originally developed as a tool to access bioactivities encoded by soil bacteria in a culture-independent, phenotype-targeted manner (5). A key advantage of this approach is that all hits are directly associated with a cloned fragment of DNA, thus permitting the identification of not only bioactive metabolites and proteins but also the genes that encode these molecules. When used to study the human microbiome, metagenomics is limited by the fact that, among potential body sites of interest, only stool generally contains sufficient bacterial biomass to permit the facile extraction and cloning of metagenomic DNA into large insert libraries. Another potential limitation of constructing a library directly from human-derived samples is that bacteria are not evenly distributed in nature, and therefore effectors from organisms that make up only a small fraction of the microbiome can be difficult to sample using this approach (6). In contrast to results in many other environments, extensive efforts to culture bacteria from the human microbiome have been quite successful. In fact, recent analyses suggest that as much as 60% to 70% of the human-associated bacteria can now be cultured, providing a means to readily access the genetic material from the majority of the microbiota (7–11). Here, we bring forward the concept of using a genomic library created from a defined collection of cultured bacteria (i.e., a multigenomic library) as a way of identifying microbiota-encoded effectors. This approach allows both the normalization of input DNA and the use of DNA from bacteria found in body sites from which sufficient biomass is difficult to obtain. The resulting library is expected to contain a more balanced representation of source bacteria and therefore a more manageable resource for use in identifying effector genes.

In this study, we use a 13,300-member multigenomic cosmid library to identify effectors encoded by a collection of 116 human-associated bacterial strains found at diverse sites across the human body. Cosmid clones created using a normalized mixture of genomic DNA isolated from these strains and hosted in Escherichia coli were screened for the production of effectors that activate nuclear factor-κB (NF-κB) signaling in human cells. We selected NF-κB activation as a target phenotype because of the key role this pathway plays in diverse biological systems that are expected to be associated with the human microbiome, for example, immune homeostasis, pathogenesis, and inflammation (2, 4). Downstream effects of NF-κB signaling range from proinflammatory responses to protective functions (12), making NF-κB a potentially useful reporter for a broad range of human-associated bacterial effectors. The 21 NF-κB active cosmid clones identified represent 17 unique genomic regions from 16 different input organisms. The proteins encoded by specific effector genes identified from both commensal and pathogenic species include domains of unknown function, membrane transporters, cell wall hydrolases, and lipopolysaccharide (LPS) core biosynthetic genes. A multigenomic approach provides a simplified function-first method for exploring human microbiome genetic information, one that takes advantage of growing available resources in culturing and sequencing members of the human microbiome, which should facilitate the discovery of novel bacterial effectors from human-associated bacteria.

## RESULTS

### Construction and analysis of a multigenomic library from 116 human-associated bacteria.

**FIG 1:**
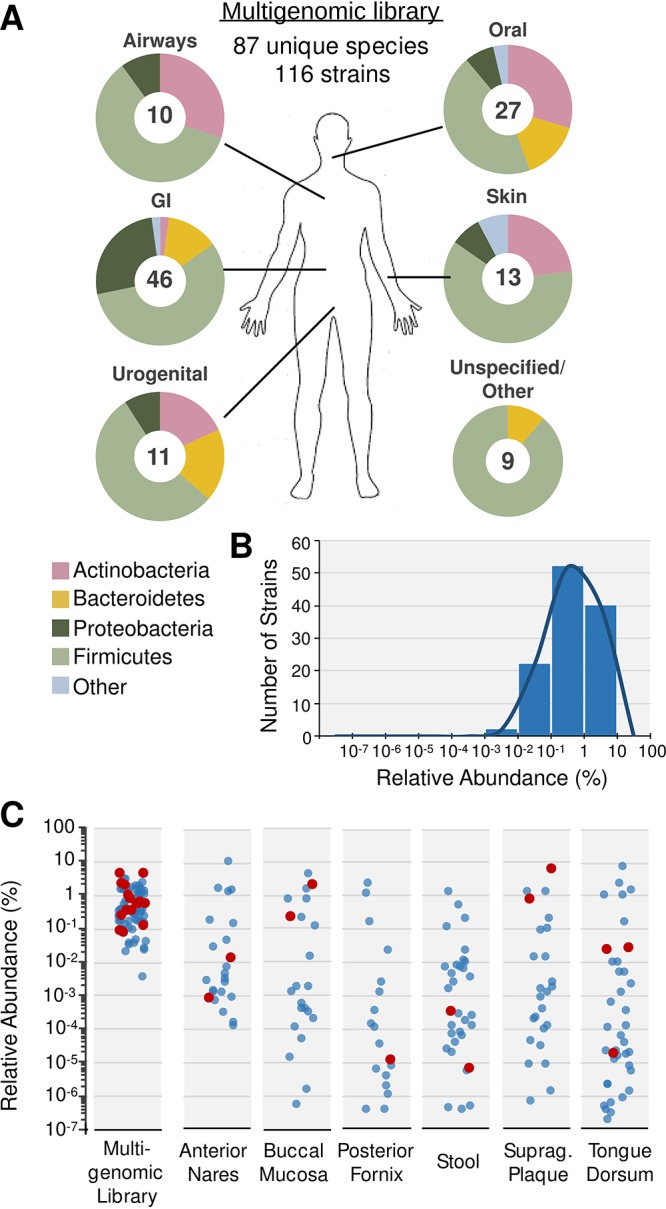
Multigenomic library from human-associated bacteria collection. (A) Taxonomic composition of the multigenomic library containing genomic DNA from 116 different strains (87 different species) according to the body site from which they were isolated and indicating the number of strains from each site (GI, gastrointestinal). (B) Distribution of strains according to their relative abundances in the multigenomic library. (C) Relative abundances of individual strains in the multigenomic library compared to the relative abundances of 46 of these strains reported in metagenomic sequences from 380 samples across different body sites in healthy human subjects (13). Red circles represent strains from which bacterial effectors were identified in our screening.

A multigenomic cosmid library was constructed from genomic DNA of 116 bacterial strains found in the Human Microbiome Project (HMP) catalog (Fig. 1A and Table S1 in the supplemental material). In total, the strains used for library construction represented 87 unique species across the four main phyla commonly observed in the human microbiome. This collection included isolates that appear at different body sites and with diverse relative abundances in the microbiota of healthy individuals (13). We did not deliberately bias our selection of strains based on any previous reports of biological activity. Based on an estimated average insert size of 30 kb per cosmid, the library comprises ∼400 Mb of multigenomic DNA, which corresponds to approximately 1-fold coverage of the 116 input genomes.

10.1128/mBio.02587-19.5 TABLE S1 List of strains from Biodefense and Emerging Infections Research Resources Repository (BEI Resources) from which genomic DNA was used in the construction of the multigenomic library. Footnotes indicate GenBank taxonomic denomination (when divergent from BEI catalog). Download Table S1, PDF file, 0.1 MB. Copyright © 2019 Estrela et al. 2019 Estrela et al. This content is distributed under the terms of the Creative Commons Attribution 4.0 International license.

To determine the abundance of each input genome in the library, all library clones were individually cultured in LB medium, and cosmid DNA was isolated from an equal volume pooling of these cultures. The collective pool of cosmids was sequenced using Illumina MiSeq 300-bp paired-end read technology, and individual sequencing reads were aligned to the genomes of the strains used to create the multigenomic library. Percent abundance based on the number of mapped reads was calculated for each strain. As expected for a normalized library containing 116 different strains, the relative abundances of the input genomes center just below 1%, with the abundance of most genomes falling within 1 order of magnitude above or below this expected value (Fig. 1B). The relative abundances of 46 of the strains included in our multigenomic library have been mapped across multiple body sites in hundreds of metagenomic samples from healthy humans (13). In this analysis, these same strains appear at relative abundances spanning almost 8 orders of magnitude (Fig. 1C), making it difficult to access the genomes of many of these strains in a metagenomic library constructed from any of the hundreds of samples sequenced in this study.

### Screening of the multigenomic library for NF-κB inducing activity.

**FIG 2:**
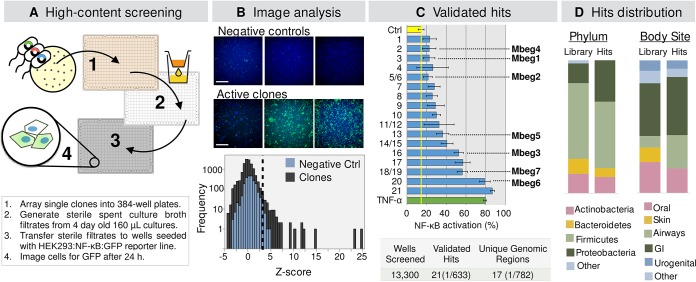
Functional screening of a multigenomic library. (A) Outline of the screening procedure. (B) Representative images of HEK293:NF-κB GFP reporter cells incubated with negative-control supernatants or active clones. Scale bar, 350 μm. The histogram shows activation *Z*-scores for all 13,300 clones screened, calculated relative to values for negative-control wells on each plate. The dashed line indicates the *Z*-score cutoff of 3. (C) Range of bioactivity level of validated hits. TNF-α (100 ng/ml) is included as a positive control. Analysis output is the percentage of GFP-positive cells (NF-κB active) to total cells, counted using nuclear staining on a per well basis. Plot shows means ± standard deviations from eight replicates. (D) Composition of the multigenomic library and the subset of clones corresponding to validated hits, according to phylum and body site (GI, gastrointestinal).

A schematic of the high-throughput protocol we used to screen the multigenomic library for NF-κB-inducing activities is depicted in Fig. 2A. Briefly, filter-sterilized culture broth from individually grown clones was transferred onto HEK293 cells transformed with green fluorescent protein (GFP) under the control of the NF-κB consensus transcriptional response element (HEK293:NF-κB:GFP). CFU counts indicated that Escherichia coli cultures remained viable throughout the duration of the assay, indicating that extensive host cell lysis was not likely to interfere with our analysis of NF-κB activity (Fig. S1A). The normalized percentage of live GFP-positive cells in each well was assessed by fluorescence microscopy (Fig. 2B). Primary hits (GFP-positive cell *Z*-score of >3) were reassayed to remove false positives, resulting in 21 validated hits. Hits induced GFP expression in 20% to 90% of cells in a well (Fig. 2C). The average baseline activation produced by E. coli supernatant was 14%, while tumor necrosis factor alpha (TNF-α), a potent known inducer of NF-κB activation (14), induced GFP expression in 80% of cells. Assessment of propidium iodide (PI)-stained cells in all screened wells showed that less than 1% of all clones increased the ratio of PI-stained cells to total cells by more than 3 standard deviations (*Z*-score of >3) compared to levels in the empty vector control wells (Fig. S1B), indicating that most clones did not cause a general increase in reporter cell death. Moreover, no validated hit caused a dramatic change in the ratio of PI-stained (dead) cells to total cells (Fig. S1C) or in the cellular and nuclear morphology of treated HEK293:NF-κB:GFP cells (Fig. S1D).

10.1128/mBio.02587-19.1 FIG S1 Cell death assessment and initial characterization of validated hits. (A) Bacterial viability (CFU counts) of E. coli grown for 4 days at 30°C in a 384-well plate. (B) Distribution of dead cell ratio *Z*-scores for all 13,300 clones screened, calculated relative to negative-control wells on each plate. Dashed line indicates *Z*-score of 3. (C) Cell death assessment (percent propidium iodide-stained cells relative to total nuclei) in each of the validated hits assayed in eight replicates. (D) Representative images (100× objective) of HEK293:NF-κB:GFP reporter cell line stained with Hoechst 33342 (nuclear stain) after 24-h treatment with supernatants from selected validated hits. Scale bar, 35 μm. (E) Bioactivity of low-molecular-weight (<10 kDa) components in sterile spent culture broth from validated hits. Sterile spent broth from 4-day-grown cultures were passed through centrifugal filter with 10-kDa molecular-weight-cutoff membrane. Bioactivity of the broth and the flowthrough was measured using NF-κB reporter assay. TNF-α (17.5 kDa) was included as a control. (F) Extracellular protein levels in sterile spent supernatants of validated hits. (G) Insert size, source genome, and body site of original isolate of the 17 unique genomic regions identified in 21 validated hits. Genomic regions that appeared in more than one clone (>80% overlap) are grouped. Download FIG S1, PDF file, 0.8 MB. Copyright © 2019 Estrela et al. 2019 Estrela et al. This content is distributed under the terms of the Creative Commons Attribution 4.0 International license.

As an initial step in the characterization of the validated active clones, we sought to determine whether the bioactive mediators they produced comprised high-molecular-weight components (e.g., proteins) or small molecules. For this experiment, sterile spent culture broth generated from each hit was passed through a 10-kDa molecular-weight-cutoff (MWCO) membrane, and the flowthrough in each case was assayed for NF-κB inducing activity. In all cases, the 10-kDa MWCO flowthrough continued to induce GFP expression, suggesting that low-molecular-weight products (<10 kDa) were responsible for the observed activities (Fig. S1E). No correlation was observed between the level of extracellular protein in sterile spent culture and NF-κB activity (Fig. S1F), suggesting that the observed NF-κB activities were not artifacts resulting from different levels of heterologous protein expression by some clones.

### Sequencing of NF-κB–inducing clones and identification of bacterial effector genes.

Cosmid DNA was extracted from each hit and Sanger sequenced using primers that flank the pJWC1 cloning site. The resulting sequences were then aligned to the reference genomes of the 116 strains comprising the multigenomic library to identify the genomic fragment in each cosmid clone. Of the 21 hits, we identified 17 unique genomic regions from 16 input strains (Fig. S1G and Table S2). This corresponds to a hit rate of one unique effector for every 782 clones screened (Fig. 2C), which represents an approximately 4-fold improvement compared to rates with previous metagenomic library screening efforts (2). The genomic regions encoding molecules that activated the NF-κB reporter arise from a distribution of major taxa that closely resembles that used in the construction of the multigenomic library (Fig. 2D). In fact, genomic fragments identified as hits arise from representatives of all major phyla included in the normalized library. As seen in other studies (2, 15), E. coli was able to express genes from a taxonomically diverse group of bacteria making it an appealing host for functional metagenomic screening studies. Effectors arise from bacteria found at most body sites represented in the library. Bacteria from the skin comprised the only group that failed to generate any effector genes. The small number of skin species examined in this initial study will need to be expanded before we can determine if this is a stochastic sampling effect or if the microbial community found in the human skin does indeed differ from communities of other body sites in regard to the production of NF-κB-inducing effectors.

10.1128/mBio.02587-19.6 TABLE S2 Predicted proteins encoded by each insert in 21 validated hits. Full-length sequences were obtained by Sanger sequencing both ends of the inserts and aligning to the reference genomes comprising the multigenomic library. Each insert was subjected to gene calling using Metagenemark, and individual ORFs were searched against NCBI nr database using Blastx. Effector genes identified by transposon mutagenesis and/or subcloning are highlighted in red. Download Table S2, PDF file, 2.3 MB. Copyright © 2019 Estrela et al. 2019 Estrela et al. This content is distributed under the terms of the Creative Commons Attribution 4.0 International license.

Seven unique clones with diverse levels of potency were subjected to *in vitro* transposon mutagenesis to identify the specific genes responsible for conferring on E. coli the ability to induce the NF-κB:GFP reporter. Cosmids randomly mutagenized by *in vitro* Tn*5* transposon mutagenesis were transformed into E. coli, and the resulting transformants were assayed for NF-κB-inducing activity. Cosmids recovered from E. coli strains that no longer induced GFP expression were sequenced. Genes found to contain transposons were identified, and their wild-type versions were subcloned into an inducible expression vector and tested for the ability to confer NF-κB-inducing activity on E. coli. Genes or sets of genes that when subcloned were found to confer on E. coli the ability to induce NF-κB were termed “microbiome bacteria effector genes,” or here, Mbegs (Table S3). The Mbegs we identified are predicted to encode proteins that fall into four functional categories: (i) proteins of unknown function (1 clone, 1 unique genomic region); (ii) cell wall hydrolases (4 clones, 3 unique genomic regions); (iii) membrane transporters (2 clones, 2 unique genomic regions); and (iv) LPS core biosynthesis (3 clones, 2 unique genomic regions).

10.1128/mBio.02587-19.7 TABLE S3 Summary of bacterial effectors identified by functional screening of a multigenomic library. The predicted protein sequence encoded by each effector gene identified was aligned to the nonredundant (nr) protein database on NCBI using blastp, and the most similar protein is recorded with the respective accession number, percent identity, and E value. The conserved domain(s) present in each of the effector proteins is presented, as analyzed by an NCBI Conserved Domain search and HMM scan. Download Table S3, PDF file, 0.02 MB. Copyright © 2019 Estrela et al. 2019 Estrela et al. This content is distributed under the terms of the Creative Commons Attribution 4.0 International license.

**FIG 3:**
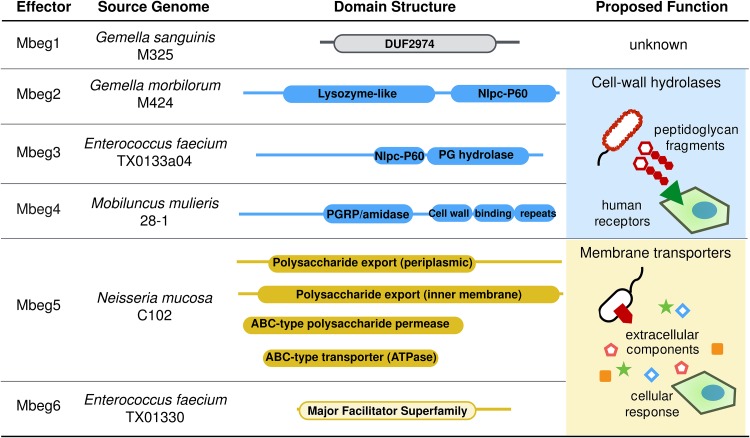
Bacterial effectors identified in six bioactive multigenomic clones. According to their domain composition, determined by NCBI conserved domains search and HMM scan, these effectors belonged to three functional categories: a protein of unknown function, cell wall hydrolases (blue), and membrane transporters (yellow).

The protein of unknown function contains a conserved domain (DUF2974, or PF11187) belonging to the alpha/beta hydrolase superfamily (16) and is encoded by an effector gene (Mbeg1) identified in a clone from Gemella sanguinis M325 (Fig. 3). G. sanguinis is a rare but emerging opportunistic pathogen that was originally isolated from blood (17) and is associated with rare cases of endocarditis (18, 19). The species has also been identified in healthy proximal intestine samples from humans (20).

In three clones, the identified effector genes encode cell wall hydrolases (Mbeg2 from Gemella morbillorum M424, Mbeg3 from Enterococcus faecium TX0133a04, and Mbeg4 from Mobiluncus mulieris 28-1) (Fig. 3). Mbeg2 contains two conserved hydrolase domains: one lysozyme-like domain, and one Nlpc/P60 family domain. The Nlpc/P60 peptidase domain is also present in the protein encoded by Mbeg3, whereas Mbeg4 contains a peptidoglycan recognition domain with an amidase catalytic site, followed by three repeats of a cell wall binding motif. Related hydrolases were identified in a previous NF-κB reporter screening from a human stool metagenomic library (2).

Two different transporter systems were identified as effectors responsible for NF-κB-inducing activity (Fig. 3). Mbeg5 from Neisseria mucosa C102 was identified as a four-gene cluster corresponding to the conserved capsule transport operon (*ctr*), which encodes the machinery for exporting capsule polysaccharide (CPS) in encapsulated meningococcal groups. CPS serves several functions in Gram-negative bacteria, including immune evasion by human pathogens (21). Mbeg6, a single open reading frame (ORF) from Enterococcus faecium TX1330, is predicted to encode major facilitator superfamily 1 (MFS-1) protein. MFS-1 transporters are known to function in bacterial adaptation to the natural host environment, exporting antimicrobial agents and virulence factors involved in colonization and infection (22). The specific molecules that are exported as a result of heterologous expression of these systems in E. coli remain to be characterized.

### LPS operon characterization.

**FIG 4:**
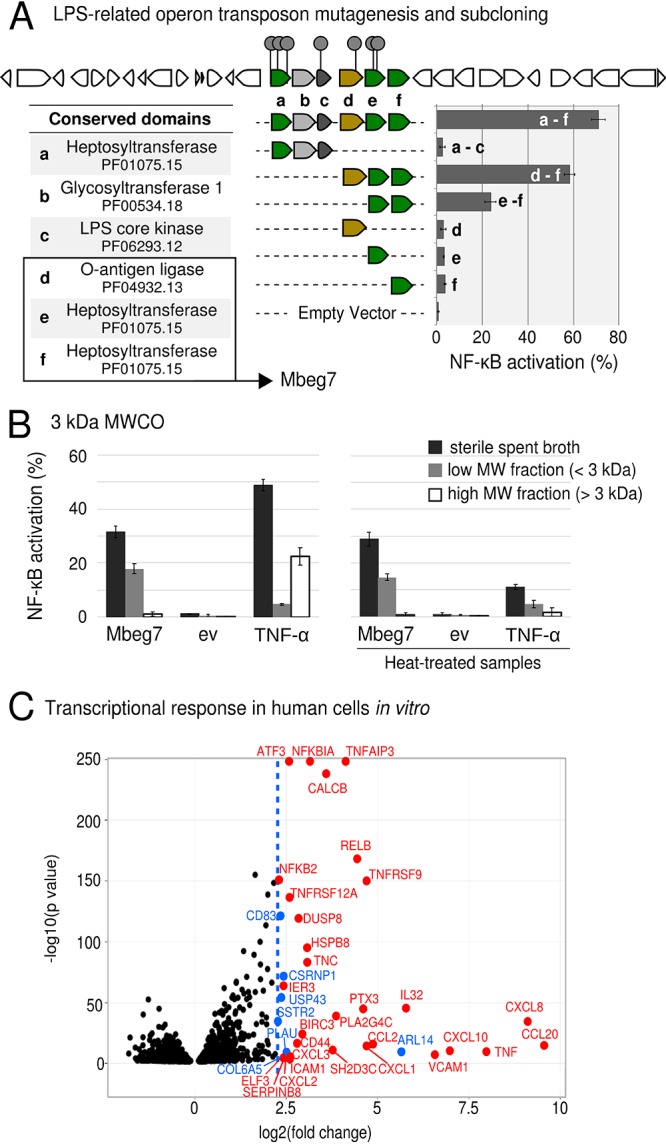
Bacterial effectors related to LPS core biosynthesis (Mbeg7). (A) A six-gene operon within the 32-kbp insert from Citrobacter portucalensis 30_2 was identified based on the position of transposon insertions (gray circles) in loss-of-function mutants. Conserved domains present in the corresponding predicted amino acid sequences were identified by a Pfam HMM scan. The operon and individual genes were subcloned into an IPTG-inducible expression vector (pET28c), and the sterile spent culture broths were assayed for NF-κB activation. (B) Bioactivity resulting from expression of the Mbeg7 operon after filtration through a 3-kDa MWCO membrane compared to the level of an empty vector control (ev). Both low- and high-molecular weight fractions were tested on an NK-κB reporter system (left panel); an aliquot of each sample was additionally submitted to heat treatment before the assay (right panel). TNF-α (17.5-kDa protein) was used as a control. (C) Characterization of human epithelial cell transcriptional response to the Mbeg7 effector *in vitro*, assessed by total mRNA sequencing analysis. The volcano plot shows genes differentially expressed (adjusted *P* value of <0.05) with a fold change higher than 5 [log_2_(foldchange) > 2.3, indicated in blue] in cells treated with the bioactive product of Mbeg7 clone versus expression in the empty vector control. Genes with known involvement in inflammation processes are indicated in red.

Two clones containing overlapping genomic regions from Citrobacter portucalensis 30_2 were among the most potent hits we observed. An operon of six genes predicted to encode lipopolysaccharide core biosynthetic proteins was identified by transposon mutagenesis as being associated with the NF-κB-inducing activity (Fig. 4A). Interestingly, a homologous operon was present in a third clone containing a genomic fragment from C. portucalensis 4_7_47_CFAA. This clone was associated with the highest level of activity we observed among our hits (Fig. 2C, clone 21). In addition to the LPS biosynthesis cluster, this clone contains a SpoT/RelA homolog predicted to encode a (p)ppGpp synthetase. The (p)ppGpp alarmone is a global regulator of gene expression in bacteria (23). SpoT/RelA homologs from *Bacteroides* were the most common NF-κB-inducing effector genes identified from human stool metagenomes (2). The presence of a second predicted effector on the same clone may explain the increased potency of the clone from C. portucalensis 4_7_47_CFAA.

The potent bioactivity presented by all three clones containing the LPS core biosynthetic operon prompted a more detailed investigation on this operon. We initially subcloned individual genes as well as subsets of genes from the six-gene LPS operon identified by transposon mutagenesis and found that three genes were necessary and sufficient for conferring NF-κB-inducing phenotype. The collection of these three genes was termed Mbeg7 (Fig. 4A, open reading frames d, e, and f). The activity produced by Mbeg7 passed through a 3-kDa MWCO filter and remained active after heat treatment (15 min at 95°C), suggesting that it was the result of a heat-stable small molecule (Fig. 4B). The three genes that make up this Mbeg are predicted to encode two LPS heptosyltransferase domain-containing proteins and one ligase, which is predicted to attach the O-antigen chain to an LPS core. While bacterial LPS is well documented as an important pathogen-associated molecular pattern and prominent virulence factor of many Gram-negative pathogens (24), finding an LPS-related effector in our functional screening was unexpected, given that HEK293 cells are deficient in Toll-like receptors (TLRs), the main mediators of host responses to bacterial LPS (2, 25). Consistent with this reasoning, we tested the activity of LPS extracted from E. coli and confirmed that the reporter is unresponsive to this molecule (Fig. S2).

10.1128/mBio.02587-19.2 FIG S2 HEK293:NF-κB:GFP reporter system is unresponsive to extracted Escherichia coli LPS. Cells were treated with increasing concentrations of LPS extracted from E. coli serotype 055:B5 (Molecular Probes). The bioactivity of LPS-related Mbeg7 product is shown on the left. Bottom blue box corresponds to a zoomed-in representation of the data in upper blue box. Download FIG S2, PDF file, 0.1 MB. Copyright © 2019 Estrela et al. 2019 Estrela et al. This content is distributed under the terms of the Creative Commons Attribution 4.0 International license.

To further characterize the phenotype induced by E. coli transformed with Mbeg7 in human cells *in vitro*, we compared transcriptomes from HEK293:NF-κB:GFP cells treated with the bioactivity produced by E. coli transformed with Mbeg7 to those treated with E. coli containing an empty vector. To reduce background noise from components in the culture medium, the bioactivity produced by cells transformed with Mbeg7 was enriched from culture broth using XAD resin extraction and reversed-phase chromatography (Fig. S3A). The empty vector culture broth control was treated in the same manner. In cells treated with the Mbeg7-associated bioactive product, we detected 36 genes that were upregulated by 5-fold or greater (*P* < 0.05) (Fig. 4C). These genes included multiple mediators of inflammation, including the following: cytokines, chemokines, and ligands with chemotactic activity; interleukin (IL) receptors and regulators (CCL20, CXCL8, TNF, CXCL10, IL-32, CCL2, CXCL1, CXCL2, and CXCL3); tumor necrosis factor (TNF) receptors (TNFRSF9 and TNFRSF12A); TNF-induced regulators such as PTX3, CALCB, and TNFAIP3 (involved in the termination of TNF- or LPS-induced NF-κB activation); adhesion molecules implicated in inflammation (VCAM1, ICAM1, TNC, SERPINB8, SH2D3C, and CD44); elements of NF-κB signaling (RELB and NFKBIA); regulators of inflammation (ATF3, IER3, ELF3, BIRC3, PLA2G4C, HSPB8, and DUSP8). Accordingly, the gene ontology (GO) enrichment analysis identified an enrichment in a collection of GO terms related to inflammation and cellular responses to molecules of bacterial origin (Fig. S3B). Taken together, these data indicate that Mbeg7 confers on E. coli the production of a metabolite that induces an NF-κB-driven inflammation response in an *in vitro* model otherwise unresponsive to canonical LPS signaling.

10.1128/mBio.02587-19.3 FIG S3 (A) Bioactivity-guided fractionation of Mbeg7 product. Sterile spent culture broth from IPTG-induced culture of T7 express E. coli cells with pET28c-Mbeg7 was extracted with Amberlite XAD16N resin, and the bioactive component was partially isolated by flash chromatography using an HP C_18_ RediSep Rf Gold reversed-phase column (left), followed by an isocratic high-performance liquid chromatography (HPLC) method using 20% acetonitrile in water on a Waters XBridge BEH phenyl column (right). Fractions were assayed for bioactivity in the NF-κB reporter system. Representative images of GFP expression are shown. (B) Gene set enrichment analysis with topGO. Download FIG S3, PDF file, 0.5 MB. Copyright © 2019 Estrela et al. 2019 Estrela et al. This content is distributed under the terms of the Creative Commons Attribution 4.0 International license.

### Mbeg7 and LPS biosynthetic diversity in the human microbiome.

**FIG 5:**
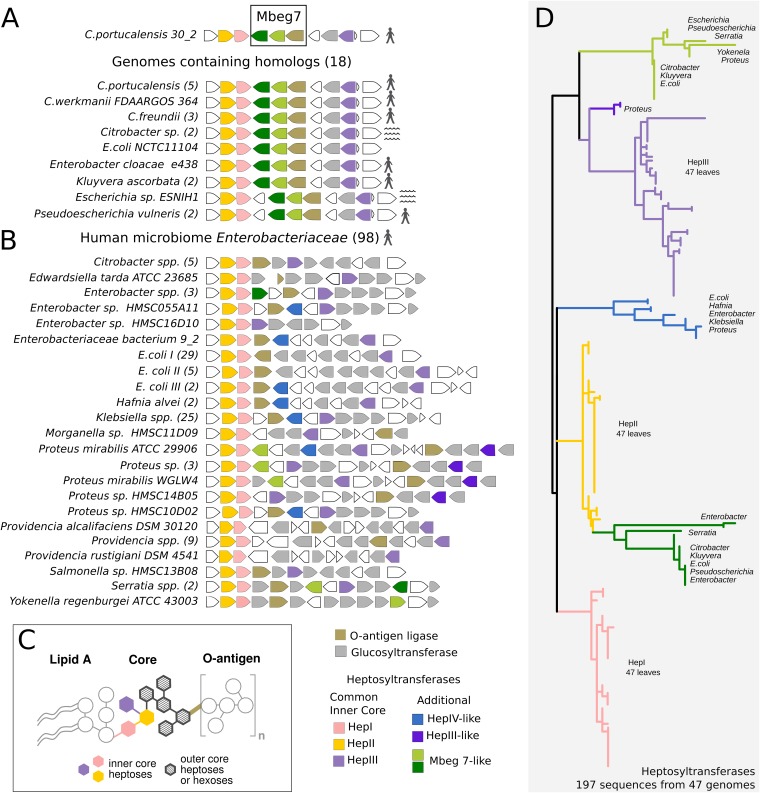
Mbeg7 from Citrobacter portucalensis corresponds to distinct features in the LPS core biosynthetic locus. LPS core biosynthetic loci identified in reference genomes of *Enterobacteriaceae* isolated from the human microbiome are shown. The 18 genomes found by blastn search to contain Mbeg7 homologues (A), plus 98 *Enterobacteriaceae* reference genomes listed in the HMP database (B), were retrieved from NCBI. The *rfa* (*waa*) locus was extracted according to source genome annotation between *kbl* and *rpmBG* genes. The number of unique sequenced genomes associated with each entry is shown in parentheses. The genomic regions were analyzed using Metagenemark and an HMM scan for functional annotation of the open reading frames according to predicted Pfam. Loci were divided in 32 groups according to the genetic organization. Aquatic-derived strains are marked with waves; human-derived strains are marked with a human figure. (C) Cartoon of LPS structure. The three conserved heptoses are color coded to match the conserved heptosyltransferase genes in the *rfa* (*waa*) loci shown in panels A and B. (D) Maximum likelihood tree of 197 heptosyltransferase sequences from 47 of the surveyed genomes, comprising one representative genome from each unique species in each of the groups depicted in panel A. Sequences were aligned using MUSCLE with default parameters on the Phylogeny.fr platform.

A blastn search of bacterial reference genomes in NCBI using the three genes that make up Mbeg7 identified 18 genomes from nine different *Enterobacteriaceae* species that contain this three-gene cassette (Fig. 5A and Table S4). Not all sequenced strains from these species contain the Mbeg7 genes; however, Mbeg7-containing strains from six of these species have been isolated from human samples. To better understand how Mbeg7 fits into LPS biosynthetic diversity in the human microbiome, we performed a comparative analysis of the *Enterobacteriaceae* LPS core biosynthesis locus (*rfa*, or *waa*) across the 18 genomes (9 species) harboring Mbeg7-like sequences, as well as 98 *Enterobacteriaceae* genomes available from the Human Microbiome Project database. The *rfa* (*waa*) loci from these genomes show a diversity in gene content and organization (Fig. 5B). The most conserved feature of these loci is the presence of three genes predicted to encode heptosyltransferases that are involved in the biosynthesis of the LPS inner core (*rfaC*, *rfaF*, and *rfaQ* encoding HepI, HepII, and HepIII, respectively) (Fig. 5C). In a phylogenetic tree of *rfa* (*waa*) locus heptosyltransferases, these gene families fall into distinct monophyletic clades (Fig. 5D). The additional heptosyltransferases encoded by Mbeg7 are phylogenetically distinct from the common heptosyltransferases (Fig. 5D). A similar result is found in a multiple-sequence alignment of O-antigen ligases encoded by Mbeg7 (Fig. S4). These phylogenetic differences suggest that enzymes encoded by Mbeg7 have different substrate specificities than other LPS biosynthesis enzymes and are therefore likely to be involved in the production of a unique LPS core structure.

10.1128/mBio.02587-19.4 FIG S4 *Enterobacteriaceae* O-antigen ligases phylogeny. Maximum likelihood tree of 47 O-antigen ligase sequences from 47 reference genomes of human-associated *Enterobacteriaceae*, comprising one representative genome from each unique species in each of the groups depicted in Fig. 5A. Sequences were aligned using MUSCLE with default parameters on the Phylogeny.fr platform and visualized using the online tool interactive Tree of Life (iTOL). Green-shaded clade is composed of homologs of the ligase encoded by one of the genes in Mbeg7. Download FIG S4, PDF file, 0.2 MB. Copyright © 2019 Estrela et al. 2019 Estrela et al. This content is distributed under the terms of the Creative Commons Attribution 4.0 International license.

10.1128/mBio.02587-19.8 TABLE S4 blastn search querying the three effector genes in Mbeg7 against bacterial reference genomes on NCBI. Download Table S4, PDF file, 0.1 MB. Copyright © 2019 Estrela et al. 2019 Estrela et al. This content is distributed under the terms of the Creative Commons Attribution 4.0 International license.

## DISCUSSION

Functional screening of the multigenomic library that we generated from 116 distinct human associated-bacteria identified 17 unique genomic regions that confer on E. coli the ability to induce NF-κB signaling. Based on the redundancy rate we observed (four redundant genomic regions in a total of 21 hits), we expect that future studies would benefit from using libraries that exceed 1-fold coverage of the input DNA. Ultimately, the optimal size for a multigenomic library will depend on a number of features including, among others, the number of input genomes, the size of the input genomes, and the accuracy in normalizing the input DNA.

The specific effector genes (i.e., Mbegs) identified from the seven bioactive clones we analyzed in detail fell into diverse functional categories. These Mbegs represent starting points for more in-depth studies on the mechanisms by which human-associated bacteria interact with the host. Although it is possible that, in some cases, NF-κB induction may arise from stress responses generated by E. coli, previous studies suggest that this is not generally the case for this assay (2). For example, the cell wall hydrolases, found in three hits from this study as well as those identified in previous metagenomic screening efforts (2), likely represent a common strategy by which bacteria generate metabolites that are recognized by the human host. Peptidoglycan is known to interact with host signaling in diverse ways. For example, human cells can sense bacterial cell wall components and their breakdown products via pattern-recognition receptors (e.g., NOD1 and NOD2) that lead to activation of NF-κB-mediated transcription (26, 27). Modification of peptidoglycans is also a common mechanism employed by bacteria to interfere with host signaling (28). A bacterial peptidoglycan hydrolase of the NlpC/P60 family has also been shown to play a role in commensally induced protection against enteric infections by promoting host epithelial barrier function (29, 30). While cell wall hydrolases have been previously associated with host-microbiota interactions, the DUF2974 domain-containing protein of unknown function that is encoded by Mbeg1 is the first example of this protein family being associated with a potential effector function in the human microbiome.

While the specific LPS-related structure encoded by bacteria containing the Mbeg7 effector has not yet been determined, it is notable that, in the context of human-associated bacteria, specific modifications of LPS structure can have major effects in the microbially induced modulation of host processes such as autoimmunity (31), proliferation and differentiation of colonic epithelial cells (32), and host tolerance of gut microbes (33). Structural variation in the oligosaccharide core region of LPS has also been implicated in pathogenicity (34, 35). Studies on LPS signaling and its role in pathogenesis have largely focused on either the lipid A fragment or the highly variable O-antigen chain. The role of LPS core oligosaccharide variability has been less extensively studied (36, 37). The consequences of additional heptosyltransferase genes in the LPS core biosynthetic locus is not known in the context of bacterial pathogenicity and host-microbe interactions; however, there is mounting evidence that bacterial heptose derivatives and biosynthetic intermediates act as important signaling molecules in microbial recognition and host immune responses, particularly via TLR-independent pathways (38–42). Although it remains to be seen how exactly LPS biosynthesis differs in strains harboring Mbeg7, we suspect that modified LPS-related structures may induce a strong proinflammatory response in human cells and potentially represent a distinct mechanism by which this select group of organisms interacts with the human host.

Within the multigenomic library, the genomes that yielded the 21 validated hits appeared in a relatively narrow range of relative abundances (Fig. 1C, red circles). In metagenomic samples from across diverse body sites of healthy humans, these same genomes appear at relative abundances spanning 7 orders of magnitude. The large fraction of low-frequency species that is seen in the human microbiome could only be sampled from a natural metagenome using an impractically large library construction and screening campaign. For instance, Enterococcus faecium TX1330 makes up on average 0.00033% of the bacteria in healthy human stool (13). The strain was present in our library at a relative abundance of 0.33%. In order to have the same chance of identifying the E. faecium-derived bioactivity we found in the multigenomic library (Mbeg6), a stool metagenomic library would need to be 1,000-fold larger (i.e., 13 million clones). The compact nature of a multigenomic library should afford the more facile exploration of diverse heterologous hosts and reporter assays, and we expect that these factors will increase the rate at which novel bacterial effectors are discovered from the human microbiome.

## MATERIALS AND METHODS

### Genomic DNA, library construction, and sequencing.

Purified genomic DNA from 116 human-associated bacterial strains was purchased from the Biodefense and Emerging Infections Research Resources Repository (BEI Resources) (see Table S1 in the supplemental material). All samples were analyzed by electrophoresis (0.7% agarose) and were verified to contain chromosomal DNA fragments of ≥20 kb. A pool was generated by combining 0.25 μg of each genomic DNA sample, according to the concentration provided by the manufacturer. DNA from the pooled sample was precipitated using isopropanol and resuspended in 40 μl of nuclease-free water (Millipore Sigma, Burlington, MA) at a final DNA concentration of 125 μg/ml. The multigenomic DNA pool was blunt ended (End-It DNA End-Repair kit; Lucigen, Middleton, WI) and ligated (Fast-Link DNA Ligation kit; Lucigen) into ScaI-digested pJWC1 cosmid vector (43). Cosmids were packaged into lambda phage (MaxPlax Lambda Packaging Extracts; Lucigen) and transfected into Escherichia coli EC100 cells in the presence of 10 mM MgSO_4_. Transformants were selected by tetracycline resistance and SacB sucrose sensitivity on LB agar plates containing tetracycline (15 μg/ml) and sucrose (10%). A total of 13,300 individual clones were robotically arrayed into 50 384-well plates and stored at –80°C as glycerol stocks. Cultures of individual clones grown overnight at 37°C in 160 μl of LB medium with 15 μg/ml tetracycline were combined, and cosmids were purified from cell pellet (total ∼1 g of wet weight) using a NucleoBond Xtra Midi kit (Macherey-Nagel, Düren, Germany) according to the manufacturer’s instructions. The sample was sequenced using an Illumina MiSeq instrument, and the 300-bp paired-end reads were processed by the seqtk trimfq toolkit using the default settings (https://github.com/lh3/seqtk). Quality-trimmed reads that aligned to pJWC1 sequence or to E. coli K-12 substrain MG1655 were removed from analysis to account for vector-derived sequences and host genomic DNA contamination, respectively. The remaining reads were aligned to 116 genomes comprising the multigenomic library using Bowtie2 (44). Because of the high similarity between genomes at the strain level, in cases when multiple strains of a given species were included in the library, reads were averaged among all input strains of each species. For nine of the strains, the genome was not published at the time of this analysis, and therefore the representative genome of the species was used for mapping.

### Reporter cell line and culture conditions.

The NF-κB reporter cell line (2) consisted of HEK293-TN cells stably transfected with the pGreenFire lentiviral NF-κB GFP-luciferase plasmid (Systems Biosciences, Palo Alto, CA), with a minimal cytomegalovirus (CMV) promoter and four copies of the NF-κB transcriptional response element controlling GFP expression. HEK293 cells do not express Toll-like receptor 2 (TLR2) and TLR4, thereby reducing baseline activation by E. coli host membrane components. Cells were routinely cultured in 75-cm^2^ flasks at 37°C and 5% CO_2_ in Dulbecco’s modified Eagle’s medium (DMEM) supplemented with l-glutamine (200 mM), penicillin (100 U/ml), streptomycin (100 U/ml), 10% fetal bovine serum (FBS), and phenol red (15.9 mg/liter). Within 18 h prior to each assay, cells were trypsinized, suspended in phenol red-free DMEM supplemented as above, and counted using trypan blue staining and a Countess automated cell counter (Invitrogen). Cells were seeded on cell culture-grade clear-bottom, black 384-well plates (Corning, New York, NY) at 2,500 cells/well in 25 μl of phenol red-free DMEM.

### NF-κB bioactivity screening, hit validation, and initial characterization.

Individual multigenomic library clones arrayed into 384-well plate wells were cultivated for 4 days at 30°C in 160 μl LB medium with 15 μg/ml tetracycline. E. coli cell viability under the growth conditions we used for screening was determined based on counts of CFU that appeared on LB agar plates containing 15 μg/ml tetracycline. The cultures were pelleted by centrifugation at 4,000 × *g* for 30 min at 4°C, and the supernatant was filtered through a 0.2-μm-pore-size membrane (Pall, Port Washington, NY) to generate sterile spent culture broth. Using an automated liquid-handling system (Tecan EVO, Tecan, Männedorf, Switzerland), 20 μl was transferred onto cells seeded as described above. After 24 h of incubation, 10 μl of a phosphate-buffered saline (PBS) solution containing nuclear staining (2 μg/ml Hoechst 33342) and dead cell marker (6 μg/ml propidium iodide) was added to each well using a Multidrop instrument (Thermo Fisher Scientific, Waltham, MA). Images were taken with a 10× objective using an ImageXpress XLS Widefield High Content Microscope (Molecular Devices, San Jose, CA) with the fluorescent filters Texas red (excitation, 562 nm [562_ex_]; emission, 624 nm [624_em_]) for propidium iodide (PI), fluorescein isothiocyanate (FITC; 482_ex_ and 536_em_) for GFP, and 4′,6′-diamidino-2-phenylindole (DAPI; 377_ex_ and 447_em_) for Hoechst 33342 and analyzed using an automated custom module in MetaXpress software (Molecular Devices). Cell death was assessed as the ratio of PI-stained cells to total Hoechst-stained nuclei in each well. NF-κB activation was measured as the ratio of live GFP-expressing cells to total live cells in each well. Results were normalized to a set of negative-control wells in each plate (E. coli host transformed with empty pJWC1 vector) and expressed as *Z*-scores. Clones with a *Z*-score greater than 3 were assayed a second time in eight replicates under the same protocol. Hits were considered validated when they showed a *Z*-score greater than 2.5 in at least six out of the eight wells. Representative images of cells treated with supernatant from validated hits were taken with a 100× objective to assess reporter cell morphology. Supernatants were also analyzed for extracellular protein content using a Qubit fluorescent protein assay kit (Thermo Fischer Scientific) according to the manufacturer’s instructions. For molecular-weight-cutoff experiments, validated hits were inoculated in 1 ml of LB medium with 15 μg/ml tetracycline in deep-well 96-well plates, and sterile spent culture broth was generated as described above. Samples were processed through a 10-kDa MWCO Amicon centrifugal filter unit (Millipore Sigma) according to the manufacturer’s instructions, and the flowthrough was assayed for NF-κB activity as described above.

### Sequence annotation of inserts from bioactive clones and identification of effector genes by transposon mutagenesis.

Cosmid DNA was obtained from each bioactive clone using a Monarch Plasmid Miniprep kit (NEB, Ipswich, MA) and sequenced by Sanger sequencing using primers targeting the flanking regions of the ScaI site on pJWC1 vector (Table S5). Each pair of forward and reverse end sequences was submitted to a blastn search against all 116 genomes comprising the library. Insert sequences were retrieved from the corresponding source genome by determining the start and end positions from end-sequence alignment and extracting GenBank and fasta sequences between these coordinates. Extracted sequences had ORFs predicted using Metagenemark (45) and searched against the NCBI nonredundant (nr) database using blastx. Cosmid DNA from selected bioactive clones was mutagenized using the EZ-Tn*5* <KAN-2> insertion kit (Lucigen) according to the manufacturer’s instructions, desalted using agarose gel tubes, and transformed into electrocompetent E. coli EC100 cells. Mutants were selected on LB agar using kanamycin (50 μg/ml) and tetracycline (15 μg/ml), and single colonies were inoculated and assayed in quadruplicate in the NF-ĸB reporter system as described above, with the unmutagenized clone as a positive control. Knockout mutants were identified, and the locations of transposon insertions were determined by Sanger sequencing using transposon-specific sequencing primers (Lucigen).

10.1128/mBio.02587-19.9 TABLE S5 Primers used in this study. Download Table S5, PDF file, 0.02 MB. Copyright © 2019 Estrela et al. 2019 Estrela et al. This content is distributed under the terms of the Creative Commons Attribution 4.0 International license.

### Subcloning and inducible expression of effector genes.

Primers with overhanging NdeI and XhoI sequences (Table S5) were designed to PCR amplify the nucleotide sequence of effector genes of interest from the pJWC1 insert template cosmid with Q5 High-Fidelity DNA polymerase (NEB) according to the manufacturer’s protocol. The annealing temperature was calculated for each primer pair using the online NEB Tm Calculator (version 1.9.13), and extension time was set according to the expected amplicon length (30 s/kb). PCR products were gel purified and digested with NdeI and XhoI (NEB), ligated into NdeI- and XhoI-digested and dephosphorylated pET28c vector using T4 DNA ligase (NEB), and transformed into electrocompetent T7 express E. coli. Single colonies were cultured in LB medium with 50 μg/ml kanamycin, and plasmid DNA was extracted using a Monarch Plasmid Miniprep kit (NEB). Identity of the cosmid inserts was confirmed by Sanger sequencing using T7 and T7-term specific primers (Table S5). The subclones were cultured in LB medium in the presence of kanamycin (50 μg/ml) and, upon reaching an optical density at 580 nm (OD_580_) of 0.6, induced with isopropyl-β-D-thiogalactopyranoside (IPTG; 500 μM) for 20 h at 18°C. Sterile spent broth collected after this period was tested for activity using the NF-κB assay as described above.

### Extraction and bioactivity-guided fractionation.

A glycerol freezer stock of E. coli T7 Express cells with pET28c-Mbeg7 was inoculated into 50 ml of LB medium with 50 μg/ml kanamycin. The culture was incubated overnight at 37°C and 200 rpm and diluted 1:100 into 1 liter of LB medium with kanamycin. The subculture was incubated at 37°C and 200 rpm until an OD_600_ of ≈0.6 and induced with 0.5 mM IPTG from a 0.5 M IPTG stock solution. The culture was incubated at 18°C and 200 rpm for 20 h. The cells were pelleted by centrifugation (4,200 × *g* for 30 min at 4°C). The culture supernatant was filtered through 0.2-μm-pore-size bottle-top filters. Amberlite XAD16N resin (Millipore Sigma) (20 g) was added to 1 liter of supernatant in medium bottles and shaken at 80 rpm for 30 min. The resin was filtered using a bottle-top filter and washed with 1 liter of double-distilled H_2_O (ddH_2_O), and 150 ml of 50% MeOH was used to elute the extracted molecules. The eluent was dried *in vacuo*, resuspended with a small volume of 50% MeOH, and loaded onto 1 g of C_18_ silica gel (Sorbent Technologies, Norcross, GA). Flash chromatography using a Teledyne ISCO CombiFlash Rf 200 instrument equipped with a 100-g high-performance (HP) C_18_ RediSep Rf Gold column was employed for crude separation. Solvent A was water, and solvent B was acetonitrile. The method was a 0 to 60% B gradient over 30 min with a flow rate of 60 ml/min. Fractions were collected and assayed for bioactivity, and the fractions containing the highest activity were pooled and dried for further purification (Fig. S3A). Next, an isocratic high-performance liquid chromatography (HPLC) method using 20% acetonitrile in water on a Waters XBridge BEH phenyl column was designed to enrich bioactivity. The flow rate was 2.5 ml/min. Fractions were collected every 1 min, dried by evaporation, dissolved in PBS, and assayed for bioactivity. The fraction with the highest activity was dried by lyophilization. As a control, a culture of E. coli T7 Express cells transformed with pET28c empty vector was processed in parallel using the same extraction and fractionation protocol.

### Total mRNA sequencing and transcription analysis.

HEK293:NF-κB:GFP cells were grown at 37°C in 5% CO_2_ in DMEM supplemented with l-glutamine (200 mM), penicillin (100 U/ml), streptomycin (100 U/ml), 10% fetal bovine serum (FBS), and phenol red (15.9 mg/liter). Upon reaching ∼80% confluence on in 75-cm^2^ flasks, cells were trypsinized, stained with trypan blue, and counted using a Countess automated cell counter (Invitrogen, Carlsbad, CA); cells were then seeded on six-well clear tissue culture-grade plates (Corning) at 3 × 10^5^ cells/well in 4 ml of culture medium and grown for 18 h. Cells were treated with the bioactive fraction from cultures of E. coli transformed with Mbeg7 (fraction obtained as described above) or the equivalent fraction from an E. coli empty vector control (40 μl of solution in PBS, for final concentration of 10 μg/ml) and incubated for 6 h. Each condition was assayed in three separate wells in two independent experiments. Total RNA was extracted from cells in each well using a Quick-RNA kit (Zymo Research, Irvine, CA) according to the manufacturer’s protocol. High-throughput RNA sequencing (RNA-seq) libraries were generated from 100 ng of total RNA using an Illumina TruSeq Stranded mRNA LT kit. Libraries prepared with unique barcodes were pooled at equal molar ratios. The pool was denatured and sequenced on an Illumina NextSeq 500 sequencer using high-output V2 reagents and NextSeq Control Software, version 1.4, to generate 75-bp single reads, according to the manufacturer’s protocol. Genes differentially expressed in the two groups were identified with the DESeq2 package (46), using as a cutoff an adjusted *P* value of less than 0.05 and a fold change higher than 5. Differentially expressed genes (DEGs) underwent gene set enrichment tests with the topGO package (47).

### Bioinformatic analyses of effector genes.

Translated amino acid sequences of each effector gene were submitted to a conserved domain (CD) search using the CD Search tool on NCBI. Additionally, a hidden Markov model (HMM) scan (48) was performed to identify Pfam domains present in each effector. The sequences of LPS core biosynthesis-related genes of interest were submitted to blastn search against the NCBI ref_seq database for *Bacteria*. For family-wide analysis of the LPS core biosynthesis locus (*rfa* or *waa*) in human-associated *Enterobacteriaceae*, the genomes of the 18 strains found to harbor Mbeg7 homologs (Table S4), plus 98 annotated reference genomes from *Enterobacteriaceae* strains in the HMP database (www.hmpdacc.org/hmp/catalog), were surveyed. The library host, E. coli strain K-12 substrain MG1655, was also included in this analysis. The complete list of accession numbers for these strains is presented in Table S6. The nucleotide sequences found between *kbl* and *rpmBG* genes (49) were extracted as fasta files and submitted to a custom annotation pipeline using Metagenemark to identify individual ORFs and to an HMM scan to annotate genes according to the presence of Pfam domains corresponding to heptosyltransferases (PF01075.15), glucosyltransferases (PF00535.24, PF01501.18, PF00534.18, and PF13439.4), epimerase (PF01370.19), O-antigen ligase (PF04932.13), Kdo kinase (PF06293.12), HepII kinase (PF06176.9), and Kdo transferase (PF04413.14). After the genomes were grouped according to genetic organization of the *rfa* locus, a subset of 47 genomes comprising one representative genome from each unique species in each of the groups was used for phylogenetic analysis of the heptosyltransferase sequences (total of 197 sequences) (Table S6). The analysis was performed on the Phylogeny.fr platform (50), starting with multiple sequence alignment using MUSCLE (v3.8.31) with default settings for highest accuracy (51). The alignment was curated to remove ambiguous regions using Gblocks (version 0.91b) (52) with the following parameters: minimum length of a block after gap cleaning, 10; no gap positions allowed in the final alignment; rejection of all segments with contiguous nonconserved positions bigger than 8; minimum number of sequences for a flank position, 85%. A maximum likelihood phylogenetic tree was reconstructed in the PhyML program (version 3.1/3.0, approximate likelihood-ratio test [aLRT]) (53). The Whelan and Goldman (WAG) substitution model was selected assuming an estimated proportion of invariant sites (of 0.018) and four gamma-distributed rate categories to account for rate heterogeneity across sites. The gamma shape parameter was estimated directly from the data (gamma = 1.262). Reliability for the internal branch was assessed using the aLRT test (Shimodaira-Hasegawa [SH]-like) (54). Graphical representation and editing of the tree was performed using the online tool interactive Tree of Life (iTOL) (55).

10.1128/mBio.02587-19.10 TABLE S6 Genomes and sequences included in *rfa* locus analysis (Fig. 5 and Fig. S4). Groups 1 to 9 correspond to genomes included based on homology to Mbeg7. Groups 10 to 32 correspond to *Enterobacteriaceae* genomes found on the NIH Human Microbiome Project catalog (www.hmpdacc.org; accessed November 2018) and downloaded from NCBI GenBank. Download Table S6, PDF file, 0.2 MB. Copyright © 2019 Estrela et al. 2019 Estrela et al. This content is distributed under the terms of the Creative Commons Attribution 4.0 International license.
