# Supplementary material for: Functional Multigenomic Screening of Human-Associated Bacteria for NF-κB-Inducing Bioactive Effectors
Source: mBio. 2019 Nov 19;10(6):e02587-19. doi: 10.1128/mBio.02587-19 (PMC6867899; doi:10.1128/mBio.02587-19)
Supplement: TABLE S1 [file mBio.02587-19-st001.pdf]

Organism	Strain	Phylum	BEI catalog #	GenBank WGS accession
Acidaminococcus sp.[1]	D21 (aka 2_2_8)	Firmicutes	HM-81	ACGB00000000
Acinetobacter radioresistens	SK82	Proteobacteria	HM-107	ACVR00000000
Actinomyces cardiffensis[2]	F0333	Actinobacteria	HM-147	AQHZ00000000
Actinomyces gerencseriae	F0344	Actinobacteria	HM-97	
Actinomyces graevenitzii	C83	Actinobacteria	HM-236	ACRN00000000
Actinomyces israelii	F0345	Actinobacteria	HM-98	
Actinomyces odontolyticus	F0309	Actinobacteria	HM-94	ACYT00000000
Actinomyces sp.	F0338	Actinobacteria	HM-146	AEUH00000000
Actinomyces sp.	F0332	Actinobacteria	HM-90	ACUY00000000
Actinomyces viscosus	C505	Actinobacteria	HM-238	ACRE00000000
Arcobacter butzleri	JV122	Proteobacteria	HM-298	AEPT00000000
Bacteroides eggerthii	1_2_48FAA	Bacteroidetes	HM-210	ACWG00000000
Bacteroides fragilis	3_1_12	Bacteroidetes	HM-20	ABZX00000000
Bacteroides ovatus	3_8_47FAA	Bacteroidetes	HM-1222	ACWH00000000
Bacteroides sp.[3]	1_1_6	Bacteroidetes	HM-23	ACIC00000000
Bifidobacterium breve	EX336960VC19	Actinobacteria	HM-412	
Bifidobacterium sp.[4]	12_1_47BFAA	Actinobacteria	HM-30	ADCN00000000
Campylobacter upsaliensis	JV21	Proteobacteria	HM-297	AEPU00000000
Capnocytophaga sp.	Oral Taxon 329, Strain F0087	Bacteroidetes	HM-267	AFHP00000000
Citrobacter freundii [5]	4_7_47CFAA	Proteobacteria	HM-299	ACDJ00000000
Citrobacter sp.[5]	30_2	Proteobacteria	HM-34	ADLG00000000
Clostridiales bacterium [6]	3_1_39B/D5	Firmicutes	HM-84	ADBG00000000
Clostridium aldenense	WAL-18727	Firmicutes	HM-307	
Clostridium bolteae	WAL-14578	Firmicutes	HM-318	ADLI00000000
Clostridium citroniae	WAL-17108	Firmicutes	HM-315	ADLJ00000000
Clostridium citroniae	Strain WAL-19142	Firmicutes	HM-316	ADLK00000000
Clostridium clostridioforme	WAL-7855	Firmicutes	HM-317	ADLM00000000
Clostridium clostridioforme	2_1_49FAA	Firmicutes	HM-306	ADLL00000000
Clostridium difficile[7]	70-100-2010	Firmicutes	HM-745	AGAC00000000
Clostridium difficile[7]	050-P50-2011	Firmicutes	HM-747	AGAB00000000
Clostridium difficile[7]	P07 (CDC#2007054)	Firmicutes	HM-88	ADVM00000000
Clostridium difficile[7]	P08 (CDC#2007019)	Firmicutes	HM-89	ADNX00000000
Clostridium hathewayi [8]	WAL-18680	Firmicutes	HM-308	ADLN00000000
Clostridium innocuum	6_1_30	Firmicutes	HM-173	
Clostridium orbiscindens [9]	1_3_50AFAA	Firmicutes	HM-303	ADLO00000000
Clostridium perfringens	WAL-14572	Firmicutes	HM-310	ADLP00000000
Clostridium sp.	7_2_43FAA	Firmicutes	HM-36	ACDK00000000
Clostridium sp.	HGF2	Firmicutes	HM-287	AENW00000000
Clostridium sp.	MSTE9	Firmicutes	HM-764	AKFU00000000
Clostridium symbiosum	WAL-14163	Firmicutes	HM-309	ADLQ00000000
Clostridium symbiosum	WAL-14673	Firmicutes	HM-319	ADLR00000000
Corynebacterium amycolatum	SK46	Actinobacteria	HM-109	ABZU00000000
Deinococcus grandis	SK125	Deinococcus	HM-111	
Enterococcus faecalis	TX0104	Firmicutes	HM-201	ACGL00000000
Enterococcus faecalis	TUSoD Ef11	Firmicutes	HM-51	ACOX00000000
Enterococcus faecalis	TX13122	Firmicutes	HM-202	ACGM00000000
Enterococcus faecalis	HH122 (aka TX0921 and EnGen0297)	Firmicutes	HM-200	ACIX00000000
Enterococcus faecium	TX0133a04	Firmicutes	HM-463	AEBC00000000
Enterococcus faecium	TX1330 (aka SE34)	Firmicutes	HM-204	ACHL00000000
Escherichia coli	MS 85-1	Proteobacteria	HM-337	ADWQ00000000
Escherichia coli	MS 110-3	Proteobacteria	HM-343	ADTW00000000
Escherichia coli	MS 16-3	Proteobacteria	HM-345	ADUA00000000
Escherichia coli	83972 (aka ABU 83972)	Proteobacteria	HM-50	ACGN00000000
Escherichia sp.[10]	3_2_53FAA	Proteobacteria	HM-38	ACAC0000000
Finegoldia magna	SY01 (aka SY403409CC001050417)	Firmicutes	HM-293	AFUI00000000

Fusobacterium nucleatum subsp. polymorphum	F0401 (aka 112230 or Oral Clone BS019)	Fusobacteria	HM-260	ADDB00000000
Fusobacterium periodonticum	1_A_54/D10	Fusobacteria	HM-41	ACIF00000000
Gemella haemolysans	M341	Firmicutes	HM-239	ACRO00000000
Gemella morbillorum	M424	Firmicutes	HM-240	ACRX00000000
Gemella sanguinis	M325	Firmicutes	HM-241	ACRY00000000
Klebsiella sp [11]	1_1_55	Proteobacteria	HM-44	ACXA00000000
Lachnospiraceae bacterium	7_1_58FAA	Firmicutes	HM-153	ACTW00000000
Lachnospiraceae sp.	6_1_63FAA	Firmicutes	HM-150	ACTV00000000
Lachnospiraceae sp.[12]	ACC2	Firmicutes	HM-480	AGEL00000000
Lactobacillus crispatus	EX849587VC01	Firmicutes	HM-370	
Lactobacillus gasseri [13]	JV-V03	Firmicutes	HM-104	ACGO00000000
Lactobacillus gasseri	MV-122	Firmicutes	HM-644	ABWH00000000
Lactobacillus jensenii	JV-V16	Firmicutes	HM-105	CM000953
Lactobacillus reuteri	CF48-3A	Firmicutes	HM-102	ACHG00000000
Micrococcus luteus	SK58	Actinobacteria	HM-114	ADCD00000000
Mobiluncus mulieris	UPII 28-I (aka 28-I)	Actinobacteria	HM-125	ADBR00000000
Neisseria mucosa	C102	Proteobacteria	HM-242	ACRG00000000
Neisseria sp.	F0314	Proteobacteria	HM-91	ADEA00000000
Oribacterium sinus	F0268	Firmicutes	HM-13	ACKX00000000
Parabacteroides distasonis [14]	31_2	Bacteroidetes	HM-169	ACUD00000000
Parabacteroides sp.	D13 (aka 3_1_5/D13)	Bacteroidetes	HM-77	ACPW00000000
Parvimonas sp.	F0139	Firmicutes	HM-207	AFII00000000
Parvimonas sp.	Oral Taxon 393, Strain F0440	Firmicutes	HM-563	AFUS00000000
Peptoniphilus sp.	F0141	Firmicutes	HM-263	AEAA00000000
Peptoniphilus sp.	F0131	Firmicutes	HM-6	ADCS00000000
Peptoniphilus sp.	Oral Taxon 375, Strain F0436	Firmicutes	HM-567	AFUH00000000
Peptostreptococcus anaerobius	Strain UPII 653-L	Firmicutes	HM-135	ADJN00000000
Porphyromonas uenonis	UPII 60-3 (aka 60-3)	Bacteroidetes	HM-130	ACLR00000000
Prevotella amnii	Strain CRIS 21A-A	Bacteroidetes	HM-138	ADFQ00000000
Prevotella buccae	D17 (aka 3_A_6B)	Bacteroidetes	HM-45	ACRB00000000
Prevotella buccalis	CRIS 12C-C (ATCC® 35310TM)	Bacteroidetes	HM-137	ADEG00000000
Prevotella denticola	F0289	Bacteroidetes	HM-208	CP002589
Prevotella nigrescens	F0103	Bacteroidetes	HM-271	ADGJ00000000
Propionibacterium acidifaciens	Oral Taxon 191, Strain F0233	Actinobacteria	HM-8	ACVN00000000
Propionibacterium acnes	SK137	Actinobacteria	HM-1122	CP001977
Propionibacterium propionicum	F0230 (aka F0230a)2	Actinobacteria	HM-209	CP002734
Pseudomonas sp.[15]	2_1_26	Proteobacteria	HM-214	ACWU00000000
Ralstonia sp.	5_2_56FAA	Proteobacteria	HM-158	ACTT00000000
Rothia dentocariosa	M567	Actinobacteria	HM-245	ADDW00000000
Shigella sp.[16]	D9 (aka 36_3_1A)	Proteobacteria	HM-87	ACDL00000000
Staphylococcus aureus	MRSA131 (Col-131)	Firmicutes	HM-466	AECR00000000
Staphylococcus aureus	MN8	Firmicutes	HM-162	CM000952
Staphylococcus aureus	TCH70	Firmicutes	HM-139	ACHH00000000
Staphylococcus caprae[17]	C87	Firmicutes	HM-246	ACRH00000000
Staphylococcus epidermidis	SK135	Firmicutes	HM-118	ADEY00000000
Staphylococcus epidermidis	BCM0060	Firmicutes	HM-140	ACHE00000000
Staphylococcus epidermidis	W23144	Firmicutes	HM-142	ACJC00000000
Staphylococcus epidermidis	M23864:W2	Firmicutes	HM-144	ADMU00000000
Staphylococcus hominis	SK119	Firmicutes	HM-119	ACLP00000000
Staphylococcus warneri [18]	SK66	Firmicutes	HM-120	ACPZ00000000
Streptococcus anginosus	F0211	Firmicutes	HM-282	AECT00000000
Streptococcus cristatus	F0329	Firmicutes	HM-163	
Streptococcus gallolyticus	TX20005	Firmicutes	HM-272	AEEM00000000
Streptococcus intermedius	F0413	Firmicutes	HM-368	AFXO00000000
Streptococcus mitis	F0392	Firmicutes	HM-262	AFUO00000000
Streptococcus pneumoniae	TCH8431	Firmicutes	HM-145	CP001993
Streptococcus salivarius	SK126	Firmicutes	HM-121	ACLO00000000
Streptococcus sanguinis	VMC66	Firmicutes	HM-275	AEVH00000000
Treponema denticola	F0402 (aka B1)	Proteobacteria	HM-259	ADEC00000000
Veillonella sp.	3_1_44	Firmicutes	HM-64	ADCV00000000

Veillonella sp.	6_1_27	Firmicutes	HM-49	ADCW00000000

[1]*Acidaminococcus intestinis;* [2]*Shaalia cardiffensis;* [3]*Bacteroides thetaiotaomicron;* [4]*Bifidobacterium longum*; [5]*Citrobacter portucalensis*; [6]*Clostridium sp.*; [7]*Clostridioides difficile*; [8]*Hungatella hathewayi*; [9]*Flavonifactor plautii*; [10]*Escherichia coli*; [11]*Klebsiella variicola*; [12]*Stomatobaculum longum*; [13]*Lactobacillus paragasseri*; [14]*Porphyromonas sp.*; [15]*Pseudomonas aeruginosa*; [16]*Escherichia coli*; [17]*Staphylococcus capitis*; [18]*Staphylococcus warneri*, strain L37603
