# Supplementary material for: Functional Multigenomic Screening of Human-Associated Bacteria for NF-κB-Inducing Bioactive Effectors
Source: mBio. 2019 Nov 19;10(6):e02587-19. doi: 10.1128/mBio.02587-19 (PMC6867899; doi:10.1128/mBio.02587-19)
Supplement: FIG S1 [file mBio.02587-19-sf001.pdf]

A

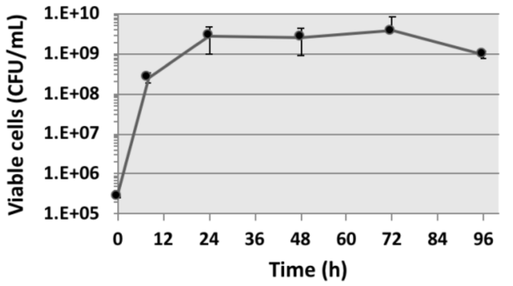

B

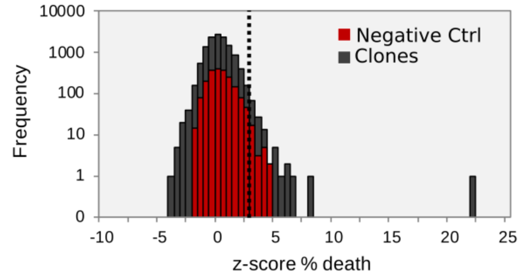

C

Hit #	Total Nuclei Ave	Total Nuclei SD	PI-stained Ave	PI-stained SD	Cell death % Ave	Cell death % SD	Cell death % z-score
Ctrl.	1815.7	239.3	10.7	2.6	0.60%	0.17%	
1	1810.8	218.0	10.7	1.3	0.59%	0.06%	-0.326
2	1956.2	209.9	11.9	3.1	0.61%	0.11%	-0.616
3	1769.6	172.5	11.7	2.8	0.65%	0.10%	-0.297
4	1728.8	288.1	11.7	1.7	0.69%	0.15%	0.5068
5/6	1889.8	240.6	11.8	2.3	0.62%	0.07%	-0.008
7	1765.4	187.0	11.3	1.8	0.64%	0.09%	0.1721
8	1771.1	162.5	9.5	0.9	0.54%	0.09%	-0.548
9	1805.9	220.6	12.1	2.9	0.68%	0.19%	1.1691
10	1790.4	201.0	13.1	1.0	0.74%	0.08%	-0.306
11/12	1714.8	211.4	9.9	2.4	0.59%	0.15%	-0.884
13	1822.3	245.3	8.5	1.4	0.47%	0.07%	-0.077
14/15	1678.1	220.1	10.8	2.2	0.64%	0.07%	-0.344
15	1844.3	216.9	10.5	0.9	0.57%	0.02%	0.2171
16	1613.9	255.7	11.1	3.2	0.68%	0.14%	-0.655
17	1887.1	190.0	12.2	1.7	0.65%	0.13%	1.0383
18/19	1894.0	249.8	11.5	2.4	0.60%	0.08%	-0.702
20	1729.9	178.0	13.6	3.6	0.79%	0.20%	-0.012
21	1688.8	181.7	17.9	4.3	1.05%	0.15%	0.3536
21	1688.8	181.7	17.9	4.3	1.05%	0.15%	0.3536

D

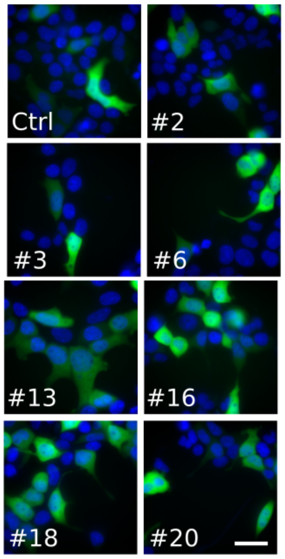

E

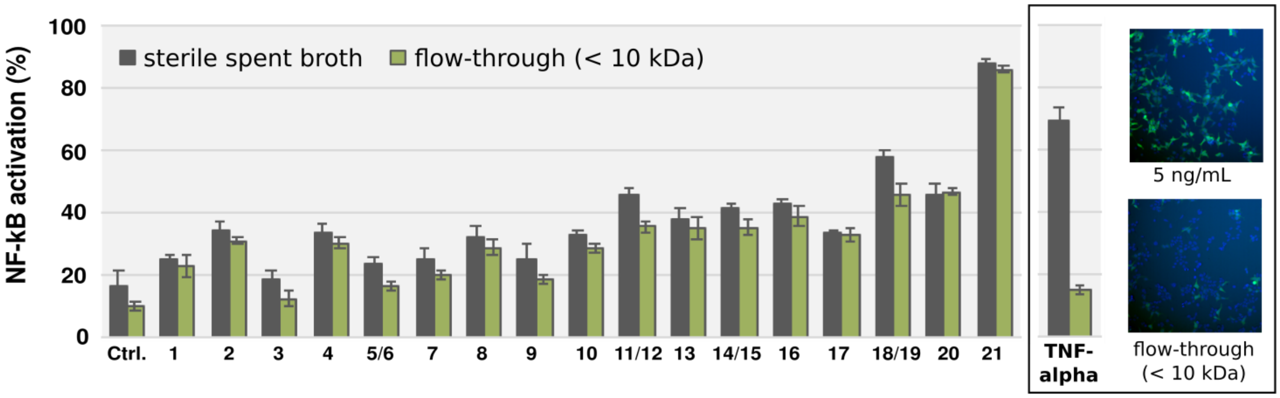

F

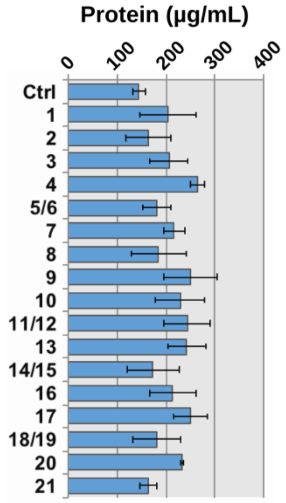

G

Hit #	Insert size (kb)	Source genome	Body site
1	33.8	*Prevotella denticola* F0289	Oral
2	29.6	*Mobiluncus mulieris* 28-1	Urogenital
3	29.9	*Gemella sanguinis* M325	Airways
4	34.8	*Neisseria sp.* oral taxon 014 str. F0314	Oral
5/6	31.8/32.8	*Gemella morbillorum* M424	Airways
7	30.7	*Clostridium symbiosum* WAL-14163	Gastrointestinal tract
8	29.8	*Rothia dentocariosa* M567	Airways
9	32.6	*Clostridium orbiscindens* 1_3_50AFAA	Gastrointestinal tract
10	14.6	*Lachnospiraceae bacterium* 7_1_58FAA	Gastrointestinal tract
11/12	34.6/28.9	*Streptococcus intermedius* F0413	Oral
13	26.9	*Neisseria mucosa* C102	Airways
14/15	34.5/32.8	*Neisseria mucosa* C102	Airways
16	26.3	*Enterococcus faecium* TX0133a04	Blood
17	31.2	*Campylobacter upsaliensis* JV21	Gastrointestinal tract
18/19	32.1/32.8	*Citrobacter portucalensis* 30_2	Gastrointestinal tract
20	31.6	*Enterococcus faecium* TX1330	Gastrointestinal tract
21	31.6	*Citrobacter portucalensis* 4_7_47CFAA	Gastrointestinal tract
