# Supplementary material for: Functional Multigenomic Screening of Human-Associated Bacteria for NF-κB-Inducing Bioactive Effectors
Source: mBio. 2019 Nov 19;10(6):e02587-19. doi: 10.1128/mBio.02587-19 (PMC6867899; doi:10.1128/mBio.02587-19)
Supplement: TABLE S2 [file mBio.02587-19-st002.pdf]

Hit #1

Gene	Length (bp)	Blast top hit description	Accession	Identity (%)	E-value
1	270	SPFH/Band 7/PHB domain protein [Prevotella melaninogenica D18]	EFC72190.1	100	0
2	126	hypothetical protein [Prevotella sp. ICM33]	WP_036922671.1	96.825	9E-81
3	317	UDP-glucose 4-epimerase [Prevotella melaninogenica]	WP_004361400.1	100	0
4	395	2-amino-3-ketobutyrate CoA ligase [Prevotella melaninogenica]	WP_004361399.1	100	0
5	880	peptidase C10 family [Prevotella melaninogenica D18]	EFC72187.1	100	0
6	216	hypothetical protein HMPREF0660_02185 [Prevotella melaninogenica D18]	EFC72186.1	100	8E-159
7	399	acyltransferase [Prevotella melaninogenica]	WP_004361396.1	100	0
8	258	transcriptional regulator [Prevotella melaninogenica]	WP_004361395.1	100	0
9	346	glycosyl transferase family 2 [Prevotella melaninogenica]	WP_004361394.1	100	0
10	128	hypothetical protein [Prevotella melaninogenica]	WP_004361393.1	100	2E-84
11	56	DNA repair protein RadC, partial [Prevotella melaninogenica]	WP_036886864.1	100	3E-29
12	626	transposase [Prevotella amnii]	WP_036857360.1	96.96	0
13	179	DNA repair protein RadC [Prevotella melaninogenica D18]	EFC71991.1	100	1E-129
14	292	hypothetical protein HMPREF0660_02396 [Prevotella melaninogenica D18]	EFC71992.1	100	0
15	106	putative FeS assembly SUF system protein [Prevotella sp. F0091]	ERJ79420.1	100	5E-69
16	254	MULTISPECIES: UDP-2,3-diacylglucosamine hydrolase [Prevotella]	WP_004361641.1	100	0
17	382	acetate kinase [Prevotella melaninogenica]	WP_004361642.1	100	0
18	347	phosphotransacetylase [Prevotella melaninogenica]	WP_036886971.1	100	0
19	611	acetyltransferase [Prevotella melaninogenica]	WP_004361644.1	100	0
20	258	membrane protein [Prevotella melaninogenica]	WP_004361646.1	100	0
21	280	dihydropteroate synthase [Prevotella melaninogenica]	WP_004361648.1	100	0
22	188	MULTISPECIES: elongation factor P [Prevotella]	WP_004361650.1	100	4E-135
23	51	MULTISPECIES: 50S ribosomal protein L34 [Prevotella]	WP_004361653.1	100	1E-26
24	259	putative transmembrane PASTA-domain protein [Prevotella melaninogenica]	WP_004361655.1	100	0
25	365	ribosomal large subunit pseudouridine synthase D [Prevotella melaninogenica]	WP_004361657.1	100	0
26	332	D-alanine--D-alanine ligase [Prevotella melaninogenica]	WP_004361659.1	100	0
27	323	peptidase [Prevotella melaninogenica]	WP_004361660.1	100	0
28	242	membrane protein [Prevotella melaninogenica DNF00666]	KGF54358.1	99.587	3E-167
29	158	arginine repressor [Prevotella melaninogenica]	WP_004361664.1	100	1E-108
30	31	MULTISPECIES: transcriptional regulator [Prevotella]	WP_013264432.1	100	1E-14

Hit #2

Gene	Length (aa)	Blast top hit description	Accession	Identity (%)	E-value
1	134	sugar ABC transporter permease [Mobiluncus mulieris]	WP_004012111.1	100	4E-90
2	468	alpha-amylase [Mobiluncus mulieris]	WP_004015631.1	100	0
3	351	LacI family transcription regulator [Mobiluncus mulieris]	WP_004012113.1	100	0
4	425	transcriptional regulator [Mobiluncus mulieris]	WP_004015633.1	100	0
5	406	transcriptional regulator [Mobiluncus mulieris]	WP_004015634.1	100	0
6	374	glycosyl transferase [Mobiluncus mulieris]	WP_039886268.1	100	0
7	156	hypothetical protein [Mobiluncus mulieris]	WP_004012117.1	99.359	1E-103
8	236	glycosyl transferase [Mobiluncus mulieris]	WP_004012118.1	99.576	3E-171
9	753	N-acetylmuramoyl-L-alanine amidase [Mobiluncus mulieris]	WP_004015637.1	100	0
10	404	glycosyl transferase [Mobiluncus mulieris]	WP_004015638.1	99.752	0
11	381	glycosyl transferase [Mobiluncus mulieris]	WP_004015639.1	99.738	0
12	472	teichoic acid transporter [Mobiluncus mulieris]	WP_004015640.1	100	0
13	374	glycosyl transferase [Mobiluncus mulieris]	WP_004015642.1	100	0
14	161	co-chaperonin GroES [Mobiluncus mulieris]	WP_004016618.1	100	8E-114
15	132	hypothetical protein [Mobiluncus mulieris]	WP_004012126.1	99.242	2E-88
16	183	hypothetical protein [Mobiluncus mulieris]	WP_004015648.1	100	2E-133
17	319	dehydrogenase [Mobiluncus mulieris]	WP_004015649.1	100	0
18	366	aminotransferase DegT [Mobiluncus mulieris]	WP_004015650.1	100	0
19	213	bacterial transferase hexapeptide repeat protein [Mobiluncus mulieris ATCC 35243]	EEJ54245.1	100	5E-150
20	333	glycosyltransferase [Mobiluncus mulieris]	WP_004015652.1	100	0
21	382	hypothetical protein [Mobiluncus mulieris]	WP_004015653.1	100	0
22	385	UDP-N-acetylglucosamine 2-epimerase [Mobiluncus mulieris]	WP_004015655.1	100	0
23	376	hypothetical protein [Mobiluncus mulieris]	WP_039886240.1	100	0
24	399	heparan-alpha-glucosaminide N-acetyltransferase [Mobiluncus mulieris]	WP_039886270.1	100	0
25	411	UDP-N-acetyl-D-mannosamine dehydrogenase [Mobiluncus mulieris]	WP_004012138.1	100	0

Hit #3

Gene	Length (aa)	Blast top hit description	Accession	Identity (%)	E-value
1	133	hypothetical protein [Gemella sanguinis]	WP_006363907.1	99.213	3E-79
2	798	hypothetical protein [Gemella sanguinis]	WP_006363906.1	100	0
3	131	hypothetical protein [Gemella sanguinis]	WP_006363905.1	100	2E-83
4	130	hypothetical protein HMPREF0433_00516 [Gemella sanguinis M325]	EGF88724.1	100	2E-83
5	127	hypothetical protein [Gemella sanguinis]	WP_006363903.1	100	2E-82
6	744	ATPase [Gemella sanguinis]	WP_006363902.1	100	0
7	662	cell division protein FtsH [Gemella sanguinis]	WP_006363901.1	100	0
8	181	hypoxanthine phosphoribosyltransferase [Gemella sanguinis]	WP_006363900.1	99.448	1E-124
9	439	tRNA(Ile)-lysidine synthetase [Gemella sanguinis]	WP_006363898.1	100	0
10	202	S1 RNA binding domain protein [Gemella sanguinis]	WP_006363897.1	100	4E-134
11	684	ATPase P [Gemella sanguinis]	WP_006363895.1	100	0
12	93	hypothetical protein [Gemella sanguinis]	WP_006363894.1	100	1E-55
13	309	tetrapyrrole methylase [Gemella sanguinis]	WP_006363893.1	100	0
14	189	SAM-dependent methyltransferase [Gemella sanguinis]	WP_006363892.1	100	3E-131
15	190	adenylate cyclase [Gemella sanguinis]	WP_006363891.1	100	8E-129
16	301	peptide ABC transporter permease [Gemella sanguinis]	WP_006363890.1	100	0
17	321	peptide ABC transporter permease [Gemella sanguinis]	WP_006363889.1	100	0
18	310	peptide ABC transporter substrate-binding protein [Gemella sanguinis]	WP_006363888.1	100	0
19	332	peptide ABC transporter ATPase [Gemella sanguinis]	WP_006363887.1	100	0
20	271	metallo-hydrolase [Gemella sanguinis]	WP_006363886.1	99.631	0
21	460	NADH oxidase [Gemella sanguinis]	WP_006363885.1	100	0
22	401	tryptophan synthase subunit alpha [Gemella sanguinis]	WP_006363884.1	100	0
23	437	amino acid permease [Gemella sanguinis]	WP_006363883.1	100	0
24	154	hypothetical protein [Gemella sanguinis]	WP_006363882.1	99.351	8E-100
25	225	hypothetical protein HMPREF0433_00495 [Gemella sanguinis M325]	EGF88703.1	100	2E-160
26	197	hypothetical protein [Gemella sanguinis]	WP_006363880.1	100	1E-137
27	59	MULTISPECIES: hypothetical protein [Gemella]	WP_003147520.1	100	4E-33
28	238	MULTISPECIES: hypothetical protein [Gemella]	WP_003147521.1	100	5E-171

Hit #4

Gene	Length (aa)	Blast top hit description	Accession	Identity (%)	E-value
1	53	thymidylate kinase [Neisseria sp. oral taxon 014]	WP_009173896.1	100	5E-26
2	331	aminodeoxychorismate lyase [Neisseria sp. oral taxon 014]	WP_009173897.1	100	0
3	186	N-acetyl-anhydromuranmyl-L-alanine amidase [Neisseria sp. oral taxon 014]	WP_009173898.1	100	4E-136
4	419	cell division protein ZipA [Neisseria sp. oral taxon 014]	WP_009173899.1	100	0
5	870	NAD-dependent DNA ligase LigA [Neisseria sp. oral taxon 014]	WP_009173900.1	100	0
6	344	Acyl-coenzyme A:6-aminopenicillanic acid acyl-transferase [Neisseria sp. oral taxon 014 str. F0314]	EFI24688.1	100	0
7	320	hypothetical protein [Neisseria sp. oral taxon 014]	WP_009173902.1	100	0
8	290	UTP--glucose-1-phosphate uridylyltransferase [Neisseria sp. oral taxon 014]	WP_009173903.1	100	0
9	485	potassium transporter Trk [Neisseria sp. oral taxon 014]	WP_009173904.1	100	0
10	91	50S ribosomal protein L31 [Neisseria sp. oral taxon 014]	WP_009173905.1	100	2E-62
11	44	hypothetical protein predicted by Glimmer/Critica [Neisseria meningitidis alpha275]	CBA05147.1	68.421	4E-09
13	163	transcription elongation factor GreB [Neisseria sp. oral taxon 014]	WP_009173906.1	100	2E-113
14	513	amidophosphoribosyltransferase [Neisseria sp. oral taxon 014]	WP_009173907.1	100	0
15	172	CvpA family protein [Neisseria sp. oral taxon 014]	WP_009173908.1	100	4E-117
16	391	cell division protein FtsN [Neisseria sp. oral taxon 014]	WP_009173909.1	100	0
17	425	folylpolyglutamate synthase [Neisseria sp. oral taxon 014]	WP_009173910.1	100	0
18	149	transcriptional regulator FolI [Neisseria sp. oral taxon 014]	WP_009173911.1	99.329	8E-102
19	119	hypothetical protein [Neisseria sp. oral taxon 014]	WP_009173912.1	100	4E-79
20	242	amino acid ABC transporter ATPase [Neisseria sp. oral taxon 014]	WP_009173913.1	100	7E-176
21	259	dimethyladenosine transferase [Neisseria sp. oral taxon 014]	WP_009173914.1	100	0
22	198	septum formation protein Maf [Neisseria sp. oral taxon 014]	WP_009173915.1	100	3E-143
23	92	hypothetical protein [Neisseria sp. oral taxon 014]	WP_009173916.1	100	2E-59
24	178	hypothetical protein [Neisseria sp. oral taxon 014]	WP_009173917.1	100	3E-125
25	459	argininosuccinate lyase [Neisseria sp. oral taxon 014]	WP_009173918.1	100	0
26	346	histidine kinase [Neisseria sp. oral taxon 014]	WP_009173919.1	100	0
27	248	amino acid ABC transporter permease [Neisseria sp. oral taxon 014]	WP_009173920.1	100	6E-174
28	450	protease [Neisseria sp. oral taxon 014]	WP_009173922.1	100	0
29	66	MULTISPECIES: hypothetical protein [Neisseria]	WP_003759971.1	100	2E-38
30	261	ABC transporter, ATP-binding protein [Neisseria mucosa ATCC 25996]	EFC89903.1	98.851	0
31	228	metal ABC transporter permease [Neisseria sp. oral taxon 014]	WP_009173923.1	100	4E-156
32	156	hypothetical protein HMPREF9016_00894 [Neisseria sp. oral taxon 014 str. F0314]	EFI24714.1	100	4E-107
33	305	membrane protein [Neisseria sp. oral taxon 014]	WP_009173925.1	100	0
34	177	hypothetical protein [Neisseria sp. oral taxon 014]	WP_039850789.1	99.394	4E-114
35	262	hypothetical protein [Neisseria sp. oral taxon 014]	WP_009173927.1	100	0
36	83	hypothetical protein [Neisseria sp. oral taxon 014]	WP_009173928.1	100	3E-52
37	271	ABC transporter substrate-binding protein [Neisseria sp. oral taxon 014]	WP_009173929.1	100	0
38	431	seryl-tRNA synthetase [Neisseria sp. oral taxon 014]	WP_009173930.1	100	0
39	233	phosphoenolpyruvate synthase [Neisseria meningitidis alpha275]	CBA09778.1	97.854	1E-165

Hit #5

Gene	Length (aa)	Blast top hit description	Accession	Identity (%)	E-value
1	289	hypothetical protein [Gemella morbillorum]	WP_004632307.1	100	0
2	208	serine peptidase [Gemella morbillorum]	WP_004632309.1	100	4E-142
3	239	metallo-beta-lactamase [Gemella morbillorum]	WP_004632311.1	100	6E-173
4	80	hypothetical protein [Gemella morbillorum]	WP_040461419.1	100	1E-46
5	415	recombinase RarA [Gemella morbillorum]	WP_004632314.1	100	0
6	300	UDP-N-acetylenolpyruvoylglucosamine reductase [Gemella morbillorum]	WP_004632316.1	100	0
7	178	hypothetical protein [Gemella morbillorum]	WP_004632318.1	100	6E-117
8	187	3-methyladenine DNA glycosylase [Gemella morbillorum]	WP_004632321.1	100	2E-135
9	1185	transcription-repair coupling factor [Gemella morbillorum]	WP_004632323.1	100	0
10	185	peptidyl-tRNA hydrolase [Gemella morbillorum]	WP_004632325.1	100	7E-130
11	316	ribose-phosphate pyrophosphokinase [Gemella morbillorum]	WP_004632328.1	100	0
12	405	serine hydroxymethyltransferase [Gemella morbillorum]	WP_004632329.1	100	0
13	104	conjugative transposon protein, partial [Streptococcus agalactiae BSU188]	EPT94776.1	100	3E-69
14	127	MULTISPECIES: hypothetical protein [Bacteria]	WP_000985015.1	100	2E-86
15	461	MULTISPECIES: cell division protein FtsK [Bacteria]		100	0
16	401	DNA replication initiation protein [Streptococcus pneumoniae]	AJD71864.1	99.751	0
17	73	MULTISPECIES: conjugal transfer protein [Bacteria]		100	5E-40
18	165	MULTISPECIES: antirestriction protein ArdA [Bacteria]		100	9E-115
19	168	MULTISPECIES: membrane protein [Bacteria]		100	1E-116
20	815	hypothetical protein HMPREF9501_00572, partial [Enterococcus faecalis TX0027]	EFT48591.1	100	0
21	725	MULTISPECIES: membrane protein [Bacteria]		99.862	0
22	333	MULTISPECIES: peptidase P60 [Bacteria]		100	0
23	311	hypothetical protein A961_1006 [Enterococcus faecalis ATCC 29212]	EJS80159.1	100	0
24	644	putative translation elongation factor G [Clostridium diff cile NAP07]	EFH13796.1	99.689	0
25	245	rRNA adenine N-6-methyltransferase, partial [Enterococcus faecium TX0133B]	EFR71084.1	100	2E-176
26	184	MULTISPECIES: transposon Tn917 resolvase [Bacilli]	WP_000576156.1	100	2E-128

Hit #6

Gene	Length (aa)	Blast top hit description	Accession	Identity (%)	E-value
1	182	hypothetical protein [Gemella morbillorum]	WP_004632301.1	100	8E-118
2	103	hypothetical protein [Gemella morbillorum]	WP_004632303.1	100	5E-63
3	181	hypothetical protein [Gemella morbillorum]	WP_004632305.1	100	5E-127
4	328	hypothetical protein [Gemella morbillorum]	WP_004632307.1	100	0
5	208	serine peptidase [Gemella morbillorum]	WP_004632309.1	100	4E-142
6	239	metallo-beta-lactamase [Gemella morbillorum]	WP_004632311.1	100	6E-173
7	80	hypothetical protein [Gemella morbillorum]	WP_040461419.1	100	1E-46
8	415	recombinase RarA [Gemella morbillorum]	WP_004632314.1	100	0
9	300	UDP-N-acetylenolpyruvoylglucosamine reductase [Gemella morbillorum]	WP_004632316.1	100	0
10	178	hypothetical protein [Gemella morbillorum]	WP_004632318.1	100	6E-117
11	187	3-methyladenine DNA glycosylase [Gemella morbillorum]	WP_004632321.1	100	2E-135
12	1185	transcription-repair coupling factor [Gemella morbillorum]	WP_004632323.1	100	0
13	185	peptidyl-tRNA hydrolase [Gemella morbillorum]	WP_004632325.1	100	7E-130
14	316	ribose-phosphate pyrophosphokinase [Gemella morbillorum]	WP_004632328.1	100	0
15	405	serine hydroxymethyltransferase [Gemella morbillorum]	WP_004632329.1	100	0
16	104	conjugative transposon protein, partial [Streptococcus agalactiae BSU188]	EPT94776.1	100	3E-69
17	127	MULTISPECIES: hypothetical protein [Bacteria]	WP_000985015.1	100	2E-86
18	461	MULTISPECIES: cell division protein FtsK [Bacteria]		100	0
19	401	DNA replication initiation protein [Streptococcus pneumoniae]	AJD71864.1	99.751	0
20	73	MULTISPECIES: conjugal transfer protein [Bacteria]		100	5E-40
21	165	MULTISPECIES: antirestriction protein ArdA [Bacteria]		100	9E-115
22	168	MULTISPECIES: membrane protein [Bacteria]		100	1E-116
23	815	hypothetical protein HMPREF9501_00572, partial [Enterococcus faecalis TX0027]	EFT48591.1	100	0
24	725	MULTISPECIES: membrane protein [Bacteria]		99.862	0
25	333	MULTISPECIES: peptidase P60 [Bacteria]		100	0
26	311	hypothetical protein A961_1006 [Enterococcus faecalis ATCC 29212]	EJS80159.1	100	0
27	644	putative translation elongation factor G [Clostridium diff cile NAP07]	EFH13796.1	99.689	0
28	245	rRNA adenine N-6-methyltransferase, partial [Enterococcus faecium TX0133B]	EFR71084.1	100	2E-176
29	184	MULTISPECIES: transposon Tn917 resolvase [Bacilli]	WP_000576156.1	100	2E-128
30	22	transposase [Streptococcus pneumoniae]	CFQ31250.1	100	0.00004

Hit #7

Gene	Length (aa)	Blast top hit description	Accession	Identity (%)	E-value
1	170	D-ribose transporter ATP binding protein [Clostridium sp. 7_3_54FAA]	WP_009299047.1	100	6E-115
2	137	MULTISPECIES: hypothetical protein [Clostridiales]	WP_003504625.1	100	1E-93
3	465	hypothetical protein [[Clostridium] symbiosum]	WP_003504627.1	100	0
4	114	hypothetical protein CLOSYM_00379 [[[Clostridium] symbiosum ATCC 14940]	ERI80297.1	100	9E-65
5	270	enoyl-CoA hydratase [[Clostridium] symbiosum]	WP_003504631.1	100	0
6	281	transporter [[Clostridium] symbiosum]	WP_003504632.1	100	0
7	346	daunorubicin resistance ABC transporter ATPase subunit [[[Clostridium] symbiosum WAL-14163]	EGA91844.1	100	0
8	223	transcriptional regulator, MarR family [[[Clostridium] symbiosum ATCC 14940]	ERI79563.1	98.655	2E-158
9	184	MULTISPECIES: hypothetical protein [Clostridiales]	WP_003504638.1	100	3E-121
10	193	hypothetical protein HMPREF1020_04696 [Clostridium sp. 7_3_54FAA]	EHF03344.1	99.482	6E-138
11	243	hypothetical protein [[Clostridium] symbiosum]	WP_003504643.1	100	2E-175
12	130	hypothetical protein CLOSYM_00813 [[[Clostridium] symbiosum ATCC 14940]	ERI79559.1	97.692	6E-88
13	93	MULTISPECIES: hypothetical protein [Clostridiales]	WP_003504647.1	100	1E-60
14	329	hypothetical protein [[Clostridium] symbiosum]	WP_003504649.1	100	0
15	144	phage-associated protein [[Clostridium] symbiosum]	WP_044911507.1	100	2E-101
17	127	hypothetical protein [[Clostridium] symbiosum]	WP_044911508.1	100	2E-84
18	53	NADH dehydrogenase, partial [Promicromonosporaceae bacterium W15]	WP_036954683.1	38.095	4
19	84	hypothetical protein [[Clostridium] symbiosum]	WP_003504651.1	100	7E-53
20	130	hypothetical protein [[Clostridium] symbiosum]	WP_044911509.1	100	2E-87
21	136	hypothetical protein [[Clostridium] symbiosum]	WP_003504654.1	100	2E-84
22	293	csd2 family CRISPR-associated protein [[Clostridium] symbiosum]	WP_003504657.1	100	0
23	281	lysozyme [[Clostridium] symbiosum]	WP_003504660.1	100	0
24	164	holin [[Clostridium] symbiosum]	WP_003504661.1	100	4E-111
25	37	hypothetical protein HMPREF1020_03812 [Clostridium sp. 7_3_54FAA]	EHF04327.1	91.892	4E-16
26	132	hypothetical protein [[Clostridium] symbiosum]	WP_003504662.1	100	8E-87
27	277	hypothetical protein [[Clostridium] symbiosum]	WP_003504663.1	99.639	0
28	82	hypothetical protein [[Clostridium] symbiosum]	WP_003504664.1	100	4E-51
29	367	hypothetical protein [[Clostridium] symbiosum]	WP_003504665.1	100	0
30	569	hypothetical protein [[Clostridium] symbiosum]	WP_003504666.1	100	0
31	528	hypothetical protein [[Clostridium] symbiosum]	WP_003504667.1	100	0
32	65	hypothetical protein HMPREF9474_04297 [[[Clostridium] symbiosum WAL-14163]	EGA91864.1	100	6E-39
33	125	hypothetical protein [[Clostridium] symbiosum]	WP_003504670.1	100	7E-83
34	197	hypothetical protein [[Clostridium] symbiosum]	WP_003504672.1	100	1E-141
35	108	hypothetical protein [[Clostridium] symbiosum]	WP_003504674.1	100	1E-72
36	150	hypothetical protein [[Clostridium] symbiosum]	WP_003504675.1	100	4E-105
37	102	hypothetical protein HMPREF9474_04302 [[[Clostridium] symbiosum WAL-14163]	EGA91869.1	99.02	5E-66
38	92	DNA-packaging protein [[Clostridium] symbiosum]	WP_003504679.1	100	3E-59
39	395	hypothetical protein HMPREF9474_04304 [[[Clostridium] symbiosum WAL-14163]	EGA91871.1	100	0
40	205	caudovirus prohead protease [[[Clostridium] symbiosum WAL-14163]	EGA91872.1	99.512	2E-147
41	394	portal protein [[Clostridium] symbiosum]	WP_003504686.1	100	0
42	474	terminase [[Clostridium] symbiosum]	WP_003504688.1	100	0

Hit #8

Gene	Length (aa)	Blast top hit description	Accession	Identity (%)	E-value
1	328	signal peptidase [Rothia dentocariosa]	WP_037230864.1	100	0
2	264	ribonuclease HII [Rothia dentocariosa]	WP_004004533.1	100	0
3	111	hypothetical protein HMPREF0734_00181 [Rothia dentocariosa M567]	EFJ77137.1	100	1E-73
4	148	endonuclease [Rothia dentocariosa]	WP_004004535.1	99.167	4E-81
5	514	hypothetical protein [Rothia dentocariosa]	WP_004004536.1	99.805	0
6	477	DNA protecting protein DprA [Rothia dentocariosa ATCC 17931]	ADP41263.1	99.371	0
7	356	recombinase XerC [Rothia dentocariosa]	WP_037230868.1	100	0
8	299	ACP S-malonyltransferase [Rothia dentocariosa]	WP_004004539.1	100	0
9	341	3-oxoacyl-ACP synthase [Rothia dentocariosa]	WP_004004540.1	99.707	0
10	86	putative acyl carrier protein [Rothia dentocariosa ATCC 17931]	ADP41267.1	100	9E-51
11	422	3-oxoacyl-ACP synthase [Rothia dentocariosa]	WP_004004542.1	100	0
12	491	serine protease [Rothia dentocariosa]	WP_004004543.1	100	0
13	239	membrane protein [Rothia dentocariosa]	WP_037230871.1	99.582	8E-168
14	407	ribof avin biosynthesis protein RibD [Rothia dentocariosa]	WP_037230873.1	100	0
15	425	ribof avin synthase subunit alpha [Rothia dentocariosa]	WP_004004546.1	100	0
16	253	ribof avin biosynthesis protein RibA [Rothia dentocariosa]	WP_004004547.1	100	0
17	157	6,7-dimethyl-8-ribityllumazine synthase [Rothia dentocariosa]	WP_004004549.1	100	3E-107
18	530	anthranilate synthase subunit I [Rothia dentocariosa]	WP_004004553.1	100	0
19	227	membrane protein [Rothia dentocariosa]	WP_004004554.1	100	5E-163
20	84	membrane protein [Rothia dentocariosa]	WP_004004555.1	98.81	1E-50
21	264	indole-3-glycerol-phosphate synthase [Rothia dentocariosa]	WP_004004556.1	99.621	0
22	443	tryptophan synthase subunit beta [Rothia dentocariosa]	WP_004004562.1	100	0
23	283	tryptophan synthase subunit alpha [Rothia dentocariosa]	WP_004004563.1	100	0
24	305	prolipoprotein diacylglyceryl transferase [Rothia dentocariosa]	WP_004004564.1	100	0
25	190	hypothetical protein HMPREF0734_00203 [Rothia dentocariosa M567]	EFJ77159.1	100	1E-135
26	79	hypothetical protein [Rothia dentocariosa]	WP_037230877.1	100	1E-49
27	492	pyruvate kinase [Rothia dentocariosa]	WP_004004566.1	100	0
28	204	transcriptional regulator [Rothia dentocariosa]	WP_004004567.1	100	1E-143

Hit #9

Gene	Length (aa)	Blast top hit description	Accession	Identity (%)	E-value
1	227	phage-associated protein, HI1409 family [Clostridium sp. ATCC BAA-442]	ERI80332.1	94.521	1E-142
2	61	hypothetical protein HMPREF9460_02507 [Clostridium orbiscindens 1_3_50AFAA]	KGF54816.1	100	2E-35
3	502	hypothetical protein [Flavonifractor plautii]	WP_044941504.1	100	0
4	78	MULTISPECIES: hypothetical protein [Clostridiales]	WP_021630830.1	100	2E-47
5	45	hypothetical protein HMPREF0372_03860 [Flavonifractor plautii ATCC 29863]	EHM39197.1	100	9E-22
6	72	hypothetical protein [Flavonifractor plautii]	WP_044941506.1	100	6E-43
7	409	MULTISPECIES: hypothetical protein [Clostridiales]	WP_007495000.1	100	0
8	257	hypothetical protein [Flavonifractor plautii]	WP_044941508.1	100	6E-179
9	170	hypothetical protein [Flavonifractor plautii]	WP_007495002.1	100	1E-118
10	345	hypothetical protein [Flavonifractor plautii]	WP_044941510.1	100	0
11	78	hypothetical protein [Flavonifractor plautii]	WP_007495004.1	100	7E-48
12	162	hypothetical protein [Flavonifractor plautii]	WP_007495005.1	100	3E-112
13	144	hypothetical protein [Flavonifractor plautii]	WP_009257404.1	100	4E-102
14	136	hypothetical protein [Flavonifractor plautii]	WP_007495007.1	100	2E-94
15	150	hypothetical protein [Flavonifractor plautii]	WP_044941512.1	100	7E-106
16	53	hypothetical protein HMPREF9460_02521 [Clostridium orbiscindens 1_3_50AFAA]	KGF54830.1	98.113	5E-27
17	445	hypothetical protein [Flavonifractor plautii]	WP_044941514.1	100	0
18	158	hypothetical protein [Flavonifractor plautii]	WP_007495013.1	100	1E-111
19	134	phage portal protein [Flavonifractor plautii]	WP_007495015.1	100	2E-91
20	59	hypothetical protein [Flavonifractor plautii]	WP_007495017.1	100	2E-35
21	621	tail tape measure protein [Flavonifractor plautii]	WP_044941516.1	100	0
22	249	hypothetical protein [Flavonifractor plautii]	WP_044941518.1	100	0
23	325	hypothetical protein [Flavonifractor plautii]	WP_044941522.1	99.692	0
24	162	hypothetical protein [Flavonifractor plautii]	WP_044941523.1	99.383	1E-113
25	136	hypothetical protein [Flavonifractor plautii]	WP_044941524.1	100	4E-94
26	373	baseplate J protein [Flavonifractor plautii]	WP_044941526.1	99.732	0
27	301	hypothetical protein [Flavonifractor plautii]	WP_044941527.1	100	0
28	113	hypothetical protein [Flavonifractor plautii]	WP_044941528.1	100	2E-73
29	300	hypothetical protein, partial [Flavonifractor plautii]	WP_044941706.1	100	0
30	126	hypothetical protein [Flavonifractor plautii]	WP_044941530.1	100	6E-84
31	65	hypothetical protein [Flavonifractor plautii]	WP_044941531.1	100	1E-38
32	135	hypothetical protein [Flavonifractor plautii]	WP_044941708.1	100	7E-93
33	254	hypothetical protein HMPREF9460_04302, partial [Clostridium orbiscindens 1_3_50AFAA]	KGF51187.1	100	1E-153
34	159	holin [Flavonifractor plautii]	WP_007488214.1	100	1E-104
35	178	hypothetical protein [Flavonifractor plautii]	WP_044941538.1	100	5E-122
36	196	hypothetical protein [Flavonifractor plautii]	WP_009258861.1	100	9E-136
37	241	MULTISPECIES: hypothetical protein [Clostridiales]	WP_021630638.1	100	2E-167
38	213	hypothetical protein [Flavonifractor plautii]	WP_044941540.1	100	4E-153
39	264	hypothetical protein HMPREF9460_02544 [Clostridium orbiscindens 1_3_50AFAA]	KGF54736.1	99.621	0
40	206	hypothetical protein [Flavonifractor plautii]	WP_044941543.1	100	2E-144
41	275	hypothetical protein [Flavonifractor plautii]	WP_044941545.1	100	0
42	115	MULTISPECIES: PadR family transcriptional regulator [Clostridiales]	WP_007492128.1	100	7E-76
43	319	2-hydroxyacid dehydrogenase [Flavonifractor plautii]	WP_044941548.1	100	0
44	970	pyruvate carboxylase [Flavonifractor plautii]	WP_044941551.1	100	0

Hit #10

Gene	Length (aa)	Blast top hit description	Accession	Identity (%)	E-value
1	414	methionine-tRNA ligase [Lachnospiraceae bacterium 7_1_58FAA]	EHO31852.1	100	0
2	38	methionine--tRNA ligase [Clostridium sp. ATCC BAA-442]	WP_021632734.1	100	9E-18
3	394	potassium transporter [Flavonifractor plautii]	WP_009259673.1	100	0
4	284	Tat pathway signal protein [Flavonifractor plautii]	WP_009259674.1	100	0
5	221	multidrug ABC transporter [Flavonifractor plautii]	WP_009259675.1	100	8E-158
6	454	histidine kinase [Lachnospiraceae bacterium 7_1_58FAA]	WP_009259676.1	100	0
7	255	hydrolase TatD [Flavonifractor plautii]	WP_007488929.1	100	0
8	205	radical SAM protein [Lachnospiraceae bacterium 7_1_58FAA]	WP_009259677.1	100	3E-150
9	41	hypothetical protein HMPREF0372_00738 [Flavonifractor plautii ATCC 29863]	EHM54009.1	100	2E-20
10	183	hypothetical protein [Lachnospiraceae bacterium 7_1_58FAA]	WP_009259678.1	100	2E-131
11	169	hypothetical protein [Flavonifractor plautii]	WP_009259679.1	100	1E-118
12	95	hypothetical protein HMPREF0995_03586 [Lachnospiraceae bacterium 7_1_58FAA]	EHO31863.1	100	3E-60
13	569	ATP-dependent metallopeptidase Hf B [Lachnospiraceae bacterium 7_1_58FAA]	EHO31864.1	99.824	0
14	185	MULTISPECIES: hypoxanthine phosphoribosyltransferase [Clostridiales]	WP_007488939.1	100	5E-130
15	456	tRNA(Ile)-lysidine synthetase [Flavonifractor plautii]	WP_007488941.1	100	0
16	450	replicative DNA helicase [Lachnospiraceae bacterium 7_1_58FAA]	WP_009259682.1	100	0
17	87	MULTISPECIES: 50S ribosomal protein L9 [Clostridiales]	WP_007488943.1	100	7E-51

Hit #11

Gene	Length (aa)	Blast top hit description	Accession	Identity (%)	E-value
1	155	phosphate acetyl/butaryl transferase [Streptococcus intermedius]	WP_021002583.1	100	6E-106
2	391	adenine glycosylase [Streptococcus intermedius]	WP_003072306.1	100	0
3	115	bacterocin transport accessory protein [Streptococcus intermedius]	WP_003072308.1	100	3E-78
4	572	phosphoglucomutase [Streptococcus intermedius]	WP_003072310.1	100	0
5	187	pantothenic acid transporter pant [Streptococcus intermedius]	WP_003072313.1	100	2E-120
6	183	phosphopantothenoylcysteine decarboxylase [Streptococcus intermedius]	WP_003072314.1	100	9E-129
7	230	phosphopantothenate--cysteine ligase [Streptococcus intermedius]	WP_003072316.1	100	2E-164
8	188	hypothetical protein [Streptococcus intermedius]	WP_003072319.1	100	3E-134
9	556	formate--tetrahydrofolate ligase [Streptococcus intermedius]	WP_003072321.1	100	0
10	60	hypothetical protein HMPREF9177_00259 [Streptococcus intermedius F0413]	EHG14205.1	100	3E-35
11	58	acetyltransferase [Streptococcus constellatus]	WP_006270520.1	51.282	0.01
12	221	hypothetical protein HMPREF9177_00260 [Streptococcus intermedius F0413]	EHG14206.1	100	1E-160
13	230	MULTISPECIES: membrane protein [Streptococcus]	WP_003069464.1	100	1E-166
14	495	glucose-6-phosphate 1-dehydrogenase [Streptococcus intermedius]	WP_003072327.1	100	0
15	454	major facilitator transporter [Streptococcus intermedius]	WP_003072328.1	100	0
16	271	phosphate ABC transporter substrate-binding protein [Streptococcus intermedius]	WP_003072329.1	100	0
17	301	membrane protein [Streptococcus intermedius]	WP_003072331.1	100	0
18	46	hypothetical protein HMPREF9177_00266 [Streptococcus intermedius F0413]	EHG14212.1	100	8E-21
19	709	S1 RNA-binding protein [Streptococcus intermedius]	WP_003072335.1	100	0
20	147	sprT-like [Streptococcus intermedius]	WP_003072337.1	100	3E-103
21	90	PspC domain protein [Streptococcus intermedius SK54]	EID83204.1	100	4E-57
22	180	NUDIX domain protein [Streptococcus constellatus subsp. constellatus SK53]	EID18554.1	98.333	9E-129
23	310	HPr kinase/phosphorylase [Streptococcus intermedius]	WP_003072343.1	100	0
24	257	prolipoprotein diacylglyceryl transferase [Streptococcus intermedius]	WP_003072345.1	100	0
25	127	MULTISPECIES: general stress protein [Streptococcus]	WP_003072347.1	100	5E-82
26	155	hypothetical protein [Streptococcus intermedius]	WP_003072349.1	100	5E-102
27	97	membrane protein [Streptococcus intermedius]	WP_003072351.1	100	3E-62
28	309	peptidase family U32 [Streptococcus intermedius]	WP_003072354.1	100	0
29	428	protease [Streptococcus intermedius]	WP_003072356.1	100	0
30	553	ribonuclease J [Streptococcus intermedius]	WP_003072358.1	100	0
31	263	acetyl esterase [Streptococcus intermedius]	WP_003072359.1	100	0
32	137	hypothetical protein HMPREF9177_00282 [Streptococcus intermedius F0413]	EHG14228.1	99.27	9E-94
33	121	hypothetical protein [Streptococcus intermedius]	WP_003072368.1	100	8E-79
34	631	multidrug ABC transporter ATP-binding protein [Streptococcus intermedius]	WP_003072371.1	100	0
35	118	hypothetical protein [Streptococcus intermedius]	WP_003072372.1	100	4E-78
36	287	MutR family transcriptional regulator [Streptococcus intermedius]	WP_003072373.1	100	0
37	399	FMN adenylyltransferase [Streptococcus intermedius]	WP_003072375.1	100	0
38	76	hypothetical protein [Streptococcus sinensis]	WP_037616871.1	91.304	7E-33

Hit #12

Gene	Length (aa)	Blast top hit description	Accession	Identity (%)	E-value
1	486	phosphoglucomutase [Streptococcus intermedius]	WP_003072310.1	100	0
2	187	pantothenic acid transporter pant [Streptococcus intermedius]	WP_003072313.1	100	2E-120
3	183	phosphopantothenoylcysteine decarboxylase [Streptococcus intermedius]	WP_003072314.1	100	9E-129
4	230	phosphopantothenate--cysteine ligase [Streptococcus intermedius]	WP_003072316.1	100	2E-164
5	188	hypothetical protein [Streptococcus intermedius]	WP_003072319.1	100	3E-134
6	556	formate--tetrahydrofolate ligase [Streptococcus intermedius]	WP_003072321.1	100	0
7	60	hypothetical protein HMPREF9177_00259 [Streptococcus intermedius F0413]	EHG14205.1	100	3E-35
8	58	acetyltransferase [Streptococcus constellatus]	WP_006270520.1	51.282	0.01
9	221	hypothetical protein HMPREF9177_00260 [Streptococcus intermedius F0413]	EHG14206.1	100	1E-160
10	230	MULTISPECIES: membrane protein [Streptococcus]	WP_003069464.1	100	1E-166
11	495	glucose-6-phosphate 1-dehydrogenase [Streptococcus intermedius]	WP_003072327.1	100	0
12	454	major facilitator transporter [Streptococcus intermedius]	WP_003072328.1	100	0
13	271	phosphate ABC transporter substrate-binding protein [Streptococcus intermedius]	WP_003072329.1	100	0
14	301	membrane protein [Streptococcus intermedius]	WP_003072331.1	100	0
15	46	hypothetical protein HMPREF9177_00266 [Streptococcus intermedius F0413]	EHG14212.1	100	8E-21
16	709	S1 RNA-binding protein [Streptococcus intermedius]	WP_003072335.1	100	0
17	147	sprT-like [Streptococcus intermedius]	WP_003072337.1	100	3E-103
18	90	PspC domain protein [Streptococcus intermedius SK54]	EID83204.1	100	4E-57
19	180	NUDIX domain protein [Streptococcus constellatus subsp. constellatus SK53]	EID18554.1	98.333	9E-129
20	310	HPr kinase/phosphorylase [Streptococcus intermedius]	WP_003072343.1	100	0
21	257	prolipoprotein diacylglyceryl transferase [Streptococcus intermedius]	WP_003072345.1	100	0
22	127	MULTISPECIES: general stress protein [Streptococcus]	WP_003072347.1	100	5E-82
23	155	hypothetical protein [Streptococcus intermedius]	WP_003072349.1	100	5E-102
24	97	membrane protein [Streptococcus intermedius]	WP_003072351.1	100	3E-62
25	309	peptidase family U32 [Streptococcus intermedius]	WP_003072354.1	100	0
26	428	protease [Streptococcus intermedius]	WP_003072356.1	100	0
27	553	ribonuclease J [Streptococcus intermedius]	WP_003072358.1	100	0
28	263	acetyl esterase [Streptococcus intermedius]	WP_003072359.1	100	0
29	137	hypothetical protein HMPREF9177_00282 [Streptococcus intermedius F0413]	EHG14228.1	99.27	9E-94
30	121	hypothetical protein [Streptococcus intermedius]	WP_003072368.1	100	8E-79

Hit #13

Gene	Length (aa)	Blast top hit description	Accession	Identity (%)	E-value
1	356	D-alanine--D-alanine ligase [Neisseria subf ava]	WP_004519078.1	100	0
2	456	hypothetical protein [Neisseria mucosa]	WP_003747976.1	100	0
3	349	acetyltransferase [Neisseria mucosa]	WP_003747978.1	100	0
4	1222	glycosyl transferase [Neisseria mucosa]	WP_003747981.1	100	0
5	367	capsule biosynthesis protein CapC [Neisseria mucosa]	WP_003747983.1	100	0
6	392	MULTISPECIES: sugar ABC transporter substrate-binding protein [Neisseria]	WP_003747985.1	100	0
7	393	polysaccharide export inner-membrane protein, BexC/CtrB/KpsE family [Neisseria subf ava NJ9703]	EFC53326.1	99.237	0
8	265	sugar ABC transporter permease [Neisseria mucosa]	WP_003747988.1	100	0
9	216	MULTISPECIES: ATP-binding protein [Neisseria]	WP_003747991.1	100	2E-159
10	757	RNA-binding protein [Neisseria mucosa]	WP_003747992.1	100	0
11	113	MULTISPECIES: membrane protein [Neisseria]	WP_003683849.1	100	6E-75
12	296	LysR family transcriptional regulator [Neisseria mucosa]	WP_003747995.1	100	0
13	133	hypothetical protein [Neisseria mucosa]	WP_003747996.1	100	4E-85
14	201	hypothetical protein [Neisseria mucosa]	WP_003747998.1	100	1E-142
15	473	cysteinyl-tRNA synthetase [Neisseria mucosa]	WP_003747999.1	100	0
16	163	hypothetical protein [Neisseria mucosa]	WP_036493930.1	100	2E-113
17	384	GTPase CgtA [Neisseria mucosa]	WP_003748002.1	100	0
18	162	thioredoxin [Neisseria mucosa]	WP_003748004.1	100	1E-112
19	127	thioesterase [Neisseria mucosa]	WP_003748005.1	100	1E-87
20	120	MULTISPECIES: VacJ [Neisseria]	WP_003681812.1	100	3E-79
21	275	ABC transporter [Neisseria mucosa]	WP_003748007.1	100	0
22	105	hypothetical protein HMPREF0604_01280 [Neisseria mucosa C102]	EFV80465.1	100	5E-72
23	196	periplasmic transporter [Neisseria mucosa]	WP_003748010.1	100	1E-138
24	164	ABC transporter substrate-binding protein [Neisseria mucosa]	WP_003748012.1	100	7E-111
25	258	MULTISPECIES: ABC transporter permease [Neisseria]	WP_003748013.1	100	0
26	266	toluene ABC transporter ATP-binding protein [Neisseria mucosa]	WP_003748015.1	100	0
27	53	hypothetical protein NEIFLAOT_00346 [Neisseria f avescens NRL30031/H210]	EEG34726.1	94.34	1E-28

Hit #14

Gene	Length (aa)	Blast top hit description	Accession	Identity (%)	E-value
1	97	endonuclease [Neisseria mucosa]	WP_003745697.1	100	7E-65
2	124	hypothetical protein [Neisseria mucosa]	WP_003745698.1	100	3E-85
3	290	hypothetical protein [Neisseria mucosa]	WP_003745701.1	100	0
4	166	phosphinothricin acetyltransferase [Neisseria mucosa]	WP_003745702.1	100	1E-118
5	178	isochorismatase [Neisseria mucosa]	WP_003745704.1	100	6E-126
6	572	arginyl-tRNA synthetase [Neisseria mucosa]	WP_003745707.1	100	0
7	118	hypothetical protein [Neisseria mucosa]	WP_003745709.1	100	2E-79
8	296	membrane protein [Neisseria mucosa]	WP_003745712.1	100	0
9	115	MULTISPECIES: hypothetical protein [Neisseria]	WP_003686176.1	100	5E-75
10	250	CAAX amino protease [Neisseria mucosa]	WP_003745714.1	100	2E-180
11	132	membrane protein [Neisseria mucosa]	WP_003745716.1	99.242	3E-84
12	280	membrane protein [Neisseria mucosa]	WP_003745718.1	100	0
13	337	antifreeze protein [Neisseria mucosa]	WP_003745719.1	100	0
14	506	hypothetical protein HMPREF0604_00110 [Neisseria mucosa C102]	EFV81531.1	100	0
15	48	hypothetical protein NEISUBOT_03628 [Neisseria subf ava NJ9703]	EFC52792.1	97.917	5E-21
16	127	S-adenosylmethionine decarboxylase proenzyme [Neisseria mucosa]	WP_003745725.1	100	2E-85
17	175	hypothetical protein [Neisseria mucosa]	WP_003745728.1	100	4E-127
18	563	hypothetical protein [Neisseria mucosa]	WP_003745729.1	100	0
19	534	twin-arginine translocation pathway signal [Neisseria mucosa]	WP_003745731.1	100	0
20	212	MULTISPECIES: ketohydroxyglutarate aldolase [Neisseria]	WP_003745733.1	100	4E-147
21	611	phosphogluconate dehydratase [Neisseria mucosa]	WP_003745735.1	100	0
22	481	glucose-6-phosphate 1-dehydrogenase [Neisseria mucosa]	WP_003745737.1	100	0
23	236	6-phosphogluconolactonase [Neisseria subf ava NJ9703]	EFC52802.1	97.458	1E-167
24	323	glucokinase [Neisseria mucosa]	WP_003745741.1	100	0
25	282	transcriptional regulator [Neisseria mucosa]	WP_003745745.1	100	0
26	547	glucose-6-phosphate isomerase [Neisseria mucosa]	WP_003745746.1	100	0
27	365	membrane protein [Neisseria mucosa]	WP_003745750.1	100	0
28	204	MULTISPECIES: cadmium transporter [Neisseria]	WP_003703682.1	100	2E-136
29	231	ABC transporter ATP-binding protein [Neisseria mucosa]	WP_036493531.1	100	3E-166
30	265	MULTISPECIES: ABC transporter permease [Neisseria]	WP_003745753.1	100	0
31	614	hypothetical protein [Neisseria mucosa]	WP_003745755.1	100	0
32	325	hypothetical protein [Neisseria mucosa]	WP_003745757.1	100	0
33	596	TonB-dependent receptor [Neisseria mucosa]	WP_036493534.1	100	0

Hit #15

Gene	Length (aa)	Blast top hit description	Accession	Identity (%)	E-value
1	183	translation initiation factor IF-2 [Neisseria mucosa]	WP_003745681.1	100	2E-105
2	497	transcription termination factor NusA [Neisseria mucosa]	WP_036493524.1	100	0
3	143	ribosome maturation protein RimP [Neisseria mucosa]	WP_003745685.1	100	5E-98
4	371	phosphoserine aminotransferase [Neisseria mucosa C102]	EFV81513.1	100	0
5	397	hypothetical protein [Neisseria mucosa]	WP_003745688.1	100	0
6	165	histidine triad domain protein [Neisseria f avescens NRL30031/H210]	EEG34755.1	94.545	2E-110
7	147	ribonuclease H [Neisseria mucosa C102]	EFV81516.1	100	2E-104
8	131	hypothetical protein [Neisseria mucosa]	WP_003745696.1	100	5E-85
9	116	endonuclease [Neisseria mucosa]	WP_003745697.1	100	7E-80
10	124	hypothetical protein [Neisseria mucosa]	WP_003745698.1	100	3E-85
11	290	hypothetical protein [Neisseria mucosa]	WP_003745701.1	100	0
12	166	phosphinothricin acetyltransferase [Neisseria mucosa]	WP_003745702.1	100	1E-118
13	178	isochorismatase [Neisseria mucosa]	WP_003745704.1	100	6E-126
14	572	arginyl-tRNA synthetase [Neisseria mucosa]	WP_003745707.1	100	0
15	118	hypothetical protein [Neisseria mucosa]	WP_003745709.1	100	2E-79
16	296	membrane protein [Neisseria mucosa]	WP_003745712.1	100	0
17	115	MULTISPECIES: hypothetical protein [Neisseria]	WP_003686176.1	100	5E-75
18	250	CAAX amino protease [Neisseria mucosa]	WP_003745714.1	100	2E-180
19	132	membrane protein [Neisseria mucosa]	WP_003745716.1	99.242	3E-84
20	280	membrane protein [Neisseria mucosa]	WP_003745718.1	100	0
21	337	antifreeze protein [Neisseria mucosa]	WP_003745719.1	100	0
22	506	hypothetical protein HMPREF0604_00110 [Neisseria mucosa C102]	EFV81531.1	100	0
23	48	hypothetical protein NEISUBOT_03628 [Neisseria subf ava NJ9703]	EFC52792.1	97.917	5E-21
24	127	S-adenosylmethionine decarboxylase proenzyme [Neisseria mucosa]	WP_003745725.1	100	2E-85
25	175	hypothetical protein [Neisseria mucosa]	WP_003745728.1	100	4E-127
26	563	hypothetical protein [Neisseria mucosa]	WP_003745729.1	100	0
27	534	twin-arginine translocation pathway signal [Neisseria mucosa]	WP_003745731.1	100	0
28	212	MULTISPECIES: ketohydroxyglutarate aldolase [Neisseria]	WP_003745733.1	100	4E-147
29	611	phosphogluconate dehydratase [Neisseria mucosa]	WP_003745735.1	100	0
30	481	glucose-6-phosphate 1-dehydrogenase [Neisseria mucosa]	WP_003745737.1	100	0
31	236	6-phosphogluconolactonase [Neisseria subf ava NJ9703]	EFC52802.1	97.458	1E-167
32	323	glucokinase [Neisseria mucosa]	WP_003745741.1	100	0
33	282	transcriptional regulator [Neisseria mucosa]	WP_003745745.1	100	0
34	232	hypothetical protein NEISICOT_01552 [Neisseria sicca ATCC 29256]	EET44590.1	76.22	8E-66
35	138	glucose-6-phosphate isomerase 1 domain protein [Burkholderia pseudomallei]	KGD39779.1	40.152	1E-14
36	20	glucose-6-phosphate isomerase [Neisseria sicca ATCC 29256]	EET44591.1	100	0.00008

Hit #16

Gene	Length (aa)	Blast top hit description	Accession	Identity (%)	E-value
1	249	ribokinase [Enterococcus faecium U0317]	EFF30110.1	100	5E-175
2	131	MULTISPECIES: ribose pyranase [Enterococcus]	WP_002286295.1	100	1E-87
3	457	PTS sucrose transporter subunit IIBC [Enterococcus faecium]	WP_002301351.1	100	0
4	716	hypothetical protein (NlpC/P60 family)[Enterococcus faecium]	WP_002321454.1	100	0
5	362	aldose 1-epimerase [Enterococcus faecium TX0133a01]	EFR67672.1	100	0
6	607	permease [Enterococcus faecium]	WP_002321457.1	100	0
7	250	bacitracin ABC transporter ATP-binding protein [Enterococcus faecium]	WP_002286304.1	100	2E-180
8	120	MULTISPECIES: amino acid ABC transporter ATP-binding protein [Enterococcus]	WP_002286306.1	99.167	2E-76
9	290	membrane protein [Enterococcus faecium]	WP_002286307.1	100	0
10	162	hypothetical protein [Enterococcus faecium]	WP_002301711.1	100	3E-114
11	274	hypothetical protein [Enterococcus faecium]	WP_002301712.1	100	0
12	202	MULTISPECIES: membrane protein [Enterococcus]	WP_002286313.1	100	1E-139
13	105	MULTISPECIES: transcriptional regulator [Enterococcus]	WP_002286315.1	100	1E-68
14	242	membrane protein [Enterococcus faecium]	WP_002294613.1	100	1E-168
15	294	LysR substrate binding domain protein [Enterococcus faecium TX0133a01]	EFR67683.1	100	0
16	346	histidine kinase [Enterococcus faecium]	WP_002301716.1	100	0
17	239	response regulator receiver domain protein [Enterococcus faecium TX0133a01]	EFR67685.1	100	6E-173
18	778	sulfate ABC transporter ATP-binding protein [Enterococcus faecium]	WP_002297413.1	100	0
19	133	hypothetical protein HMPREF9524_02186 [Enterococcus faecium TX0133a01]	EFR67687.1	100	4E-90
20	491	3,4-dihydroxybenzoate decarboxylase [Enterococcus faecium]	WP_002302338.1	100	0
21	194	3-octaprenyl-4-hydroxybenzoate carboxy-lyase [Enterococcus faecium]	WP_002302336.1	100	2E-139
22	139	hypothetical protein OGW_04093 [Enterococcus faecium EnGen0004]	ELA80973.1	100	6E-96
23	135	hypothetical protein [Enterococcus faecium]	WP_002321463.1	99.259	9E-92
24	236	MULTISPECIES: hypothetical protein [Enterococcus]	WP_002286342.1	100	8E-170
25	490	multidrug transporter [Enterococcus faecium]	WP_002293204.1	100	0
26	157	MULTISPECIES: transcriptional regulator [Enterococcus]	WP_002286344.1	100	6E-109
27	32	PadR family transcriptional regulator [Enterococcus faecium]	WP_002327478.1	100	2E-13

Hit #17

Gene	Length (aa)	Blast top hit description	Accession	Identity (%)	E-value
1	35	conserved hypothetical protein [Campylobacter upsaliensis JV21]	EFU71114.1	100	1E-11
2	129	conserved hypothetical protein [Campylobacter upsaliensis JV21]	EFU71115.1	100	3E-85
3	112	alanyl-tRNA synthetase [Campylobacter upsaliensis JV21]	EFU71116.1	100	2E-71
4	186	hypothetical protein HMPREF9400_1647 [Campylobacter upsaliensis JV21]	EFU71117.1	100	3E-121
5	587	hypothetical protein [Campylobacter upsaliensis]	WP_034959139.1	100	0
6	724	DNA topoisomerase III [Campylobacter upsaliensis]	WP_004278181.1	100	0
7	245	conserved hypothetical protein [Campylobacter upsaliensis JV21]	EFU71120.1	100	1E-176
8	193	hypothetical protein [Campylobacter upsaliensis]	WP_004278183.1	100	2E-134
9	113	hypothetical protein [Campylobacter upsaliensis]	WP_004278184.1	100	4E-75
10	252	hypothetical protein [Campylobacter upsaliensis]	WP_004278185.1	100	2E-180
11	206	hypothetical protein HMPREF9400_1654 [Campylobacter upsaliensis JV21]	EFU71124.1	100	6E-146
12	76	conserved hypothetical protein [Campylobacter upsaliensis JV21]	EFU71093.1	100	2E-44
13	64	hypothetical protein [Campylobacter upsaliensis]	WP_004278188.1	98.438	7E-34
14	213	hypothetical protein [Campylobacter upsaliensis]	WP_004278189.1	100	6E-151
15	71	hypothetical protein [Campylobacter upsaliensis]	WP_004278190.1	100	6E-43
16	82	LysR family transcriptional regulator [Campylobacter upsaliensis]	WP_004278191.1	100	8E-53
17	106	hypothetical protein [Campylobacter upsaliensis]	WP_004278192.1	100	3E-66
18	176	prophage Lp1 protein 30 [Campylobacter upsaliensis JV21]	EFU71099.1	100	3E-123
19	140	hypothetical protein [Campylobacter upsaliensis]	WP_004278194.1	100	2E-93
20	120	hypothetical protein [Campylobacter upsaliensis]	WP_004278195.1	100	1E-78
21	420	ABC superfamily ATP binding cassette transporter [Campylobacter upsaliensis]	WP_004278196.1	100	0
22	82	hypothetical protein, partial [Campylobacter upsaliensis]	WP_004278197.1	100	4E-48
23	258	hypothetical protein, partial [Campylobacter upsaliensis]	WP_004278198.1	100	0
24	308	hypothetical protein [Campylobacter upsaliensis]	WP_004278199.1	100	0
25	780	hypothetical protein [Campylobacter upsaliensis]	WP_004278200.1	100	0
26	187	conjugal transfer protein [Campylobacter upsaliensis]	WP_004278201.1	100	1E-130
27	122	mannitol-1-phosphate 5-dehydrogenase [Campylobacter upsaliensis JV21]	EFU71070.1	100	2E-33
28	209	cpp33 [Campylobacter upsaliensis JV21]	EFU71071.1	100	2E-147
29	73	hypothetical protein [Campylobacter upsaliensis]	WP_004278204.1	100	2E-45
30	161	hypothetical protein [Campylobacter upsaliensis]	WP_004278205.1	100	4E-107
31	267	30S ribosomal protein S15 [Campylobacter upsaliensis JV21]	EFU71074.1	100	0
32	57	conserved hypothetical protein [Campylobacter upsaliensis JV21]	EFU71075.1	100	1E-33
33	34	hypothetical protein [Bacillus cereus]	WP_044796363.1	52	8
34	93	hypothetical protein [Campylobacter upsaliensis]	WP_004278208.1	100	1E-60
35	196	conserved hypothetical protein [Campylobacter upsaliensis JV21]	EFU71077.1	99.49	7E-136
36	370	conserved hypothetical protein [Campylobacter upsaliensis JV21]	EFU71078.1	100	0
37	71	hypothetical protein [Campylobacter upsaliensis]	WP_034959146.1	100	3E-39
38	34	adhesin [Corynebacterium jeikeium]	WP_035003110.1	46.429	7
40	86	hypothetical protein [Campylobacter upsaliensis]	WP_004278213.1	100	1E-54
41	215	hypothetical protein [Campylobacter upsaliensis]	WP_004278214.1	100	1E-152
42	378	hypothetical protein [Campylobacter upsaliensis]	WP_004278215.1	100	0
43	82	guanylate kinase [Campylobacter upsaliensis]	WP_004278216.1	100	1E-50
44	332	conserved hypothetical protein [Campylobacter upsaliensis JV21]	EFU71084.1	100	0
45	70	conserved hypothetical protein [Campylobacter upsaliensis JV21]	EFU71085.1	100	9E-41
46	217	hypothetical protein HMPREF9400_1686 [Campylobacter upsaliensis JV21]	EFU71086.1	100	6E-154
47	312	hypothetical protein [Campylobacter upsaliensis]	WP_004278222.1	100	0
48	175	hypothetical protein [Campylobacter upsaliensis]	WP_004278223.1	100	8E-119
49	112	conserved hypothetical protein [Campylobacter upsaliensis JV21]	EFU71089.1	100	2E-72
50	135	hypothetical protein [Campylobacter upsaliensis]	WP_004278225.1	100	4E-91
51	98	hypothetical protein [Campylobacter upsaliensis]	WP_004278227.1	100	3E-61

Hit #18

Gene	Length (aa)	Blast top hit description	Accession	Identity (%)	E-value
1	178	MULTISPECIES: guanylate kinase [Enterobacteriaceae]	WP_003024042.1	100	2E-128
2	559	DNA ligase [Citrobacter sp. 30_2]	WP_008786482.1	100	0
3	205	MULTISPECIES: membrane protein [Enterobacteriaceae]	WP_003024049.1	100	8E-139
4	287	MULTISPECIES: hypothetical protein [Enterobacteriaceae]	WP_003024052.1	100	0
5	238	MULTISPECIES: ribonuclease PH [Enterobacteriaceae]	WP_003024054.1	100	3E-171
6	213	MULTISPECIES: orotate phosphoribosyltransferase [Citrobacter]	WP_020996052.1	100	8E-153
7	198	MULTISPECIES: division inhibitor protein [Citrobacter]	WP_003827257.1	100	2E-139
8	152	MULTISPECIES: deoxyuridine 5'-triphosphate nucleotidohydrolase [Citrobacter]	WP_003827259.1	100	1E-105
9	407	MULTISPECIES: phosphopantothenoylcysteine decarboxylase [Citrobacter]	WP_008786479.1	100	0
10	221	UPF0758 protein yicR [Citrobacter sp. 30_2]	WP_020996053.1	100	1E-160
11	78	MULTISPECIES: 50S ribosomal protein L28 [Enterobacteriaceae]	WP_003024071.1	100	3E-49
12	55	MULTISPECIES: 50S ribosomal protein L33 [Enterobacteriaceae]	WP_003024094.1	100	3E-30
13	269	5-hydroxymethyluracil DNA glycosylase [Citrobacter sp. 30_2]	WP_008786477.1	100	0
14	159	MULTISPECIES: phosphopantetheine adenylyltransferase [Enterobacteriaceae]	WP_003024099.1	100	3E-113
15	425	3-deoxy-D-manno-octulosonic acid transferase [Citrobacter sp. 30_2]	WP_008786476.1	100	0
16	75	hypothetical protein SPAB_04621 [Salmonella enterica subsp. enterica serovar Paratyphi B str. SPB7]	ABX69934.1	59.259	4
17	343	putative lipopolysaccharide heptosyltransferase III [Citrobacter sp. 30_2]	EEH95469.2	100	0
18	374	glucosyltransferase [Citrobacter sp. 30_2]	WP_008786474.1	100	0
19	266	lipopolysaccharide core heptose(I) kinase RfaP [Citrobacter sp. 30_2]	WP_008786473.1	100	0
20	410	MULTISPECIES: hypothetical protein [Citrobacter]	WP_032941570.1	100	0
21	345	MULTISPECIES: glycosyl transferase [Citrobacter]	WP_003827303.1	100	0
22	368	hypothetical protein CSAG_03818 [Citrobacter sp. 30_2]	EEH95464.2	100	0
23	320	MULTISPECIES: ADP-heptose:LPS heptosyl transferase [Citrobacter]	WP_008786470.1	100	0
24	348	ADP-heptose:LPS heptosyl transferase [Citrobacter sp. 30_2]	WP_008786469.1	100	0
25	310	MULTISPECIES: ADP-L-glycero-D-mannoheptose-6-epimerase [Enterobacteriaceae]	WP_003827312.1	100	0
26	398	2-amino-3-ketobutyrate CoA ligase [Citrobacter sp. 30_2]	WP_008786468.1	100	0
27	341	l-threonine 3-dehydrogenase [Citrobacter sp. 30_2]	WP_008786467.1	100	0
28	228	MULTISPECIES: lipopolysaccharide biosynthesis protein [Citrobacter]	WP_008786466.1	100	2E-164
29	344	glycosyl transferase [Citrobacter sp. 30_2]	WP_008786465.1	100	0
30	311	MULTISPECIES: hypothetical protein [Enterobacteriaceae]	WP_003024138.1	99.678	0
31	427	peptidase M37 [Citrobacter sp. 30_2]	WP_008786464.1	100	0
32	589	2,3-bisphosphoglycerate-independent phosphoglycerate mutase [Citrobacter youngae ATCC 29220]	EFE06284.1	94.903	0
33	143	MULTISPECIES: hypothetical protein [Enterobacteriaceae]	WP_003024148.1	100	6E-99
34	83	MULTISPECIES: glutaredoxin [Citrobacter]	WP_003024152.1	100	4E-54
35	155	MULTISPECIES: protein-export protein SecB [Enterobacteriaceae]	WP_003024155.1	100	3E-111
36	42	NAD(P)H-dependent glycerol-3-phosphate dehydrogenase [Citrobacter freundii]	WP_003827328.1	100	3E-19

Hit #19

Gene	Length (aa)	Blast top hit description	Accession	Identity (%)	E-value
1	170	Ribonuclease PH [Escherichia coli ISC11]	CDL40586.1	100	1E-115
2	213	MULTISPECIES: orotate phosphoribosyltransferase [Citrobacter]	WP_020996052.1	100	8E-153
3	198	MULTISPECIES: division inhibitor protein [Citrobacter]	WP_003827257.1	100	2E-139
4	152	MULTISPECIES: deoxyuridine 5'-triphosphate nucleotidohydrolase [Citrobacter]	WP_003827259.1	100	1E-105
5	407	MULTISPECIES: phosphopantothenoylcysteine decarboxylase [Citrobacter]	WP_008786479.1	100	0
6	221	UPF0758 protein yicR [Citrobacter sp. 30_2]	WP_020996053.1	100	1E-160
7	78	MULTISPECIES: 50S ribosomal protein L28 [Enterobacteriaceae]	WP_003024071.1	100	3E-49
8	55	MULTISPECIES: 50S ribosomal protein L33 [Enterobacteriaceae]	WP_003024094.1	100	3E-30
9	269	5-hydroxymethyluracil DNA glycosylase [Citrobacter sp. 30_2]	WP_008786477.1	100	0
10	159	MULTISPECIES: phosphopantetheine adenylyltransferase [Enterobacteriaceae]	WP_003024099.1	100	3E-113
11	425	3-deoxy-D-manno-octulosonic acid transferase [Citrobacter sp. 30_2]	WP_008786476.1	100	0
12	75	hypothetical protein SPAB_04621 [Salmonella enterica subsp. enterica serovar Paratyphi B str. SPB7]	ABX69934.1	59.259	4
13	343	putative lipopolysaccharide heptosyltransferase III [Citrobacter sp. 30_2]	EEH95469.2	100	0
14	374	glucosyltransferase [Citrobacter sp. 30_2]	WP_008786474.1	100	0
15	266	lipopolysaccharide core heptose(I) kinase RfaP [Citrobacter sp. 30_2]	WP_008786473.1	100	0
16	410	MULTISPECIES: hypothetical protein [Citrobacter]	WP_032941570.1	100	0
17	345	MULTISPECIES: glycosyl transferase [Citrobacter]	WP_003827303.1	100	0
18	368	hypothetical protein CSAG_03818 [Citrobacter sp. 30_2]	EEH95464.2	100	0
19	320	MULTISPECIES: ADP-heptose:LPS heptosyl transferase [Citrobacter]	WP_008786470.1	100	0
20	348	ADP-heptose:LPS heptosyl transferase [Citrobacter sp. 30_2]	WP_008786469.1	100	0
21	310	MULTISPECIES: ADP-L-glycero-D-mannoheptose-6-epimerase [Enterobacteriaceae]	WP_003827312.1	100	0
22	398	2-amino-3-ketobutyrate CoA ligase [Citrobacter sp. 30_2]	WP_008786468.1	100	0
23	341	l-threonine 3-dehydrogenase [Citrobacter sp. 30_2]	WP_008786467.1	100	0
24	228	MULTISPECIES: lipopolysaccharide biosynthesis protein [Citrobacter]	WP_008786466.1	100	2E-164
25	344	glycosyl transferase [Citrobacter sp. 30_2]	WP_008786465.1	100	0
26	311	MULTISPECIES: hypothetical protein [Enterobacteriaceae]	WP_003024138.1	99.678	0
27	427	peptidase M37 [Citrobacter sp. 30_2]	WP_008786464.1	100	0
28	589	2,3-bisphosphoglycerate-independent phosphoglycerate mutase [Citrobacter youngae ATCC 29220]	EFE06284.1	94.903	0
29	143	MULTISPECIES: hypothetical protein [Enterobacteriaceae]	WP_003024148.1	100	6E-99
30	83	MULTISPECIES: glutaredoxin [Citrobacter]	WP_003024152.1	100	4E-54
31	155	MULTISPECIES: protein-export protein SecB [Enterobacteriaceae]	WP_003024155.1	100	3E-111
32	339	MULTISPECIES: NAD(P)H-dependent glycerol-3-phosphate dehydrogenase [Citrobacter]	WP_008786462.1	100	0
33	273	MULTISPECIES: serine acetyltransferase [Enterobacteriaceae]	WP_003024160.1	100	0
34	157	rRNA methyltransferase [Citrobacter sp. 30_2]	WP_008786461.1	100	3E-112
35	396	l-lactate dehydrogenase [Citrobacter sp. 30_2]	WP_008786460.1	100	0
36	130	DNA-binding transcriptional repressor LldR [Citrobacter freundii]	WP_003837649.1	100	5E-89

Hit #20

Gene	Length (aa)	Blast top hit description	Accession	Identity (%)	E-value
1	217	hypothetical protein N036_40085, partial [Enterococcus gallinarum EGD-AAK12]	ERE63901.1	100	1E-153
2	455	sodium:proton antiporter [Enterococcus faecium]	WP_002310724.1	100	0
3	198	hypothetical protein [Enterococcus faecium]	WP_002314044.1	100	2E-142
4	202	hypothetical protein [Enterococcus faecium]	WP_002314045.1	100	5E-148
5	81	hypothetical protein, partial [Enterococcus faecium]	WP_002316654.1	100	2E-52
6	121	hypothetical protein, partial [Enterococcus faecium]	WP_002316655.1	100	7E-84
7	474	hypothetical protein HMPREF0352_0992 [Enterococcus faecium TX1330]	EEI60789.1	99.789	0
8	341	LacI family transcription regulator [Enterococcus faecium]	WP_002312769.1	100	0
9	335	aldose 1-epimerase [Enterococcus faecium]	WP_002316657.1	100	0
10	221	beta-phosphoglucomutase [Enterococcus faecium]	WP_002310736.1	100	2E-157
11	764	maltose phosphorylase [Enterococcus faecium]	WP_002316658.1	100	0
12	721	PTS glucose transporter subunit IIABC [Enterococcus faecium]	WP_002310738.1	100	0
13	276	phosphatase [Enterococcus faecium]	WP_002316659.1	100	0
14	566	hypothetical protein HMPREF0352_0999 [Enterococcus faecium TX1330]	EEI60787.1	100	0
15	386	peptidase C60 [Enterococcus faecium]	WP_002314050.1	99.741	0
16	147	GCN5 family acetyltransferase [Enterococcus faecium]	WP_002310743.1	100	2E-102
17	176	phenolic acid decarboxylase padC [Enterococcus faecium]	WP_002314051.1	100	3E-125
18	184	PadR family transcriptional regulator [Enterococcus faecium]	WP_002293202.1	100	3E-129
19	157	MerR family transcriptional regulator [Enterococcus faecium]	WP_002293203.1	100	7E-109
20	511	multidrug transporter [Enterococcus faecium]	WP_025477362.1	100	0
21	236	hypothetical protein [Enterococcus faecium]	WP_002310747.1	100	5E-170
22	133	hypothetical protein HMPREF0352_1008 [Enterococcus faecium TX1330]	EEI60768.1	99.248	2E-89
23	150	hypothetical protein HMPREF0352_1009 [Enterococcus faecium TX1330]	EEI60769.1	100	1E-104
24	194	3-octaprenyl-4-hydroxybenzoate carboxy-lyase [Enterococcus faecium]	WP_002293209.1	100	2E-139
25	491	3,4-dihydroxybenzoate decarboxylase [Enterococcus faecium]	WP_002314053.1	100	0
26	140	hypothetical protein HMPREF0352_1012 [Enterococcus faecium TX1330]	EEI60772.1	100	1E-95
27	43	hypothetical protein HMPREF0352_1013, partial [Enterococcus faecium TX1330]	EEI60741.1	100	2E-20
28	778	sulfate ABC transporter ATP-binding protein [Enterococcus faecium]	WP_002314054.1	100	0

Hit #21

Gene	Length (aa)	Blast top hit description	Accession	Identity (%)	E-value
1	364	sodium/glutamate symport carrier protein GltS [Citrobacter sp. CIP 55.13]	WP_005122595.1	100	0
2	693	ATP-dependent DNA helicase RecG [Citrobacter freundii]	WP_003827244.1	100	0
3	229	tRNA methyltransferase [Citrobacter freundii]	WP_003827247.1	100	2E-169
4	704	MULTISPECIES: bifunctional (p)ppGpp synthetase II/ guanosine-3',5'-bis pyrophosphate 3'-pyrophosphohydrolase [Enterobacteriaceae]	WP_003024038.1	99.858	0
5	91	MULTISPECIES: DNA-directed RNA polymerase subunit omega [Enterobacteriaceae]		100	1E-54
6	207	MULTISPECIES: guanylate kinase [Enterobacteriaceae]	WP_003024042.1	100	5E-150
7	559	DNA ligase [Citrobacter freundii]	WP_003827252.1	100	0
8	205	MULTISPECIES: membrane protein [Enterobacteriaceae]	WP_003024049.1	100	8E-139
9	287	MULTISPECIES: hypothetical protein [Enterobacteriaceae]	WP_003024052.1	100	0
10	238	MULTISPECIES: ribonuclease PH [Enterobacteriaceae]	WP_003024054.1	100	3E-171
11	213	orotate phosphoribosyltransferase [Citrobacter freundii]	WP_003827256.1	100	1E-152
12	198	MULTISPECIES: division inhibitor protein [Citrobacter]	WP_003827257.1	100	2E-139
13	152	MULTISPECIES: deoxyuridine 5'-triphosphate nucleotidohydrolase [Citrobacter]	WP_003827259.1	100	1E-105
14	407	phosphopantothenoylcysteine decarboxylase [Citrobacter freundii]	WP_003827261.1	100	0
15	221	hypothetical protein [Citrobacter freundii]	WP_003827263.1	100	3E-160
16	78	MULTISPECIES: 50S ribosomal protein L28 [Enterobacteriaceae]	WP_003024071.1	100	3E-49
17	55	MULTISPECIES: 50S ribosomal protein L33 [Enterobacteriaceae]	WP_003024094.1	100	3E-30
18	269	5-hydroxymethyluracil DNA glycosylase [Citrobacter freundii]	WP_003827288.1	100	0
19	159	MULTISPECIES: phosphopantetheine adenylyltransferase [Enterobacteriaceae]	WP_003024099.1	100	3E-113
20	425	3-deoxy-D-manno-octulosonic acid transferase [Citrobacter freundii]	WP_003827291.1	100	0
21	355	putative lipopolysaccharide heptosyltransferase III [Citrobacter freundii 4_7_47CFAA]	EHL85435.1	99.718	0
22	374	glucosyltransferase [Citrobacter freundii]	WP_003827294.1	100	0
23	158	lipopolysaccharide core heptose(I) kinase rfaP [Citrobacter freundii 4_7_47CFAA]	EHL85437.1	100	9E-113
24	114	lipopolysaccharide core heptose(I) kinase rfaP [Citrobacter freundii 4_7_47CFAA]	EHL85438.1	100	2E-77
25	425	hypothetical protein [Citrobacter freundii]	WP_032934167.1	100	0
26	345	MULTISPECIES: glycosyl transferase [Citrobacter]	WP_003827303.1	100	0
27	367	hypothetical protein HMPREF9428_03774 [Citrobacter freundii 4_7_47CFAA]	EHL85441.1	100	0
28	320	ADP-heptose:LPS heptosyl transferase [Citrobacter freundii]	WP_003827307.1	100	0
29	348	ADP-heptose:LPS heptosyl transferase [Citrobacter freundii]	WP_003827310.1	100	0
30	310	MULTISPECIES: ADP-L-glycero-D-mannoheptose-6-epimerase [Enterobacteriaceae]	WP_003827312.1	100	0
31	398	2-amino-3-ketobutyrate CoA ligase [Citrobacter freundii]	WP_003827313.1	100	0
32	341	MULTISPECIES: l-threonine 3-dehydrogenase [Enterobacteriaceae]	WP_003024132.1	100	0
33	20	MULTISPECIES: lipopolysaccharide biosynthesis protein [Citrobacter]	WP_008786466.1	100	0.02
