# Supplementary material for: Functional Multigenomic Screening of Human-Associated Bacteria for NF-κB-Inducing Bioactive Effectors
Source: mBio. 2019 Nov 19;10(6):e02587-19. doi: 10.1128/mBio.02587-19 (PMC6867899; doi:10.1128/mBio.02587-19)
Supplement: TABLE S3 [file mBio.02587-19-st003.pdf]

Mbeg	Taxon	Protein	Blastp top hit	Identity<br>e-value	Domains<br>Pfam COG cd smart
1	*Gemella sanguinis* M325	EGF88726.1<br>hypothetical protein	WP_006363906.1<br>DUF2974 domain-containing protein [*Gemella sanguinis*]	99%<br>0.0	DUF2974
2	*Gemella morbillorum* M424	EFV35992.1<br>NlpC/P60 family protein	WP_050278213.1<br>peptidase P60 [*Streptococcus pneumoniae*]	100%<br>0.0	1. Lysozyme -Like<br>2. NlpC/P60
3	*Enterococcus faecium* TX0133a04	EFS05643.1<br>NlpC/P60 family protein	WP_002321454.1<br>hypothetical protein [*Enterococcus faecium*]	100%<br>0.0	1. NlpC/P60<br>2. Glucosaminidase/FlgJ
4	*Mobiluncus mulieris* 28-1	EEZ90146.1<br>N-acetylmuramoyl-L-alanine amidase	WP_004015637.1<br>N-acetylmuramoyl-L-alanine amidase [*Mobiluncus mulieris*]	100%<br>0.0	1. PGRP /Amidase_2<br>2. CW_binding_ (3 x)
5	*Neisseria mucosa* C102	EFV80450.1<br>CPS export outer membrane protein ctrA	WP_003747985.1<br>MULTISPECIES: polysaccharide export protein [*Neisseria*]	100%<br>0.0	Wza/Poly_export
		EFV80451.1<br>CPS inner-membrane protein ctrB	WP_003747986.1<br>MULTISPECIES: CPS transporter [*Neisseria*]	100%<br>0.0	KpsE/Wzz
		EFV80452.1<br>CPS export inner-membrane protein ctrC	WP_003747988.1<br>MULTISPECIES: ABC transporter permease [*Neisseria*]	100%<br>0.0	TagG/ABC2_membrane
		EFV80453.1<br>CPS export ATP-binding protein ctrD	WP_003747991.1<br>MULTISPECIES: ABC transporter ATP-binding protein [*Neisseria*]	100%<br>6E-161	TagH/ ABC_KpsT_Wzt/<br>AAA/ABC_tran
6	*Enterococcus faecium* TX1330	EEI60777.1<br>drug resistance MFS transporter, drug:H+ antiporter-2 family	WP_025477362.1<br>DHA2 family efflux MFS transporter permease subunit [*Enterococcus faecium*]	100%<br>0.0	MFS_1
7	*Citrobacter portucalensis.* 30_2	EEH95466.1<br>hypothetical protein	WP_008786472.1<br>MULTISPECIES: hypothetical protein [*Citrobacter portucalensis*]	100%<br>0.0	Wzy_C
		EEH95465.1<br>hypothetical protein	ALD77360.1<br>ADP-heptose--LPS heptosyltransferase II [*Citrobacter portucalensis*]	99%<br>0.0	GT1_LPS_heptosytrans/<br>RfaF/Glyco_transf_9
		EEH95464.2<br>hypothetical protein	WP_020996055.1<br>MULTISPECIES: glycosyltransferase family 9 protein [*Citrobacter portucalensis*]	100%<br>0.0	GT1_LPS_heptosytrans/<br>RfaF/Glyco_transf_9
