# Supplementary figures and images for: Functional Multigenomic Screening of Human-Associated Bacteria for NF-κB-Inducing Bioactive Effectors
Source: mBio. 2019 Nov 19;10(6):e02587-19. doi: 10.1128/mBio.02587-19 (PMC6867899; doi:10.1128/mBio.02587-19)

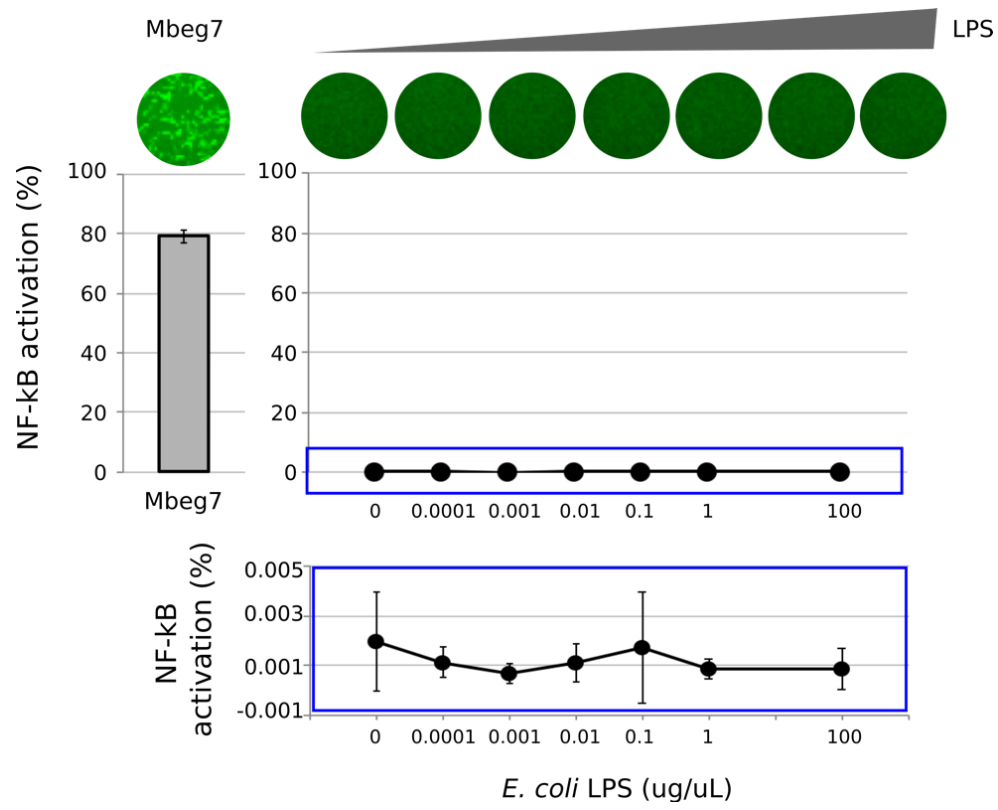

Supplement: FIG S2 [file mBio.02587-19-sf002.pdf]

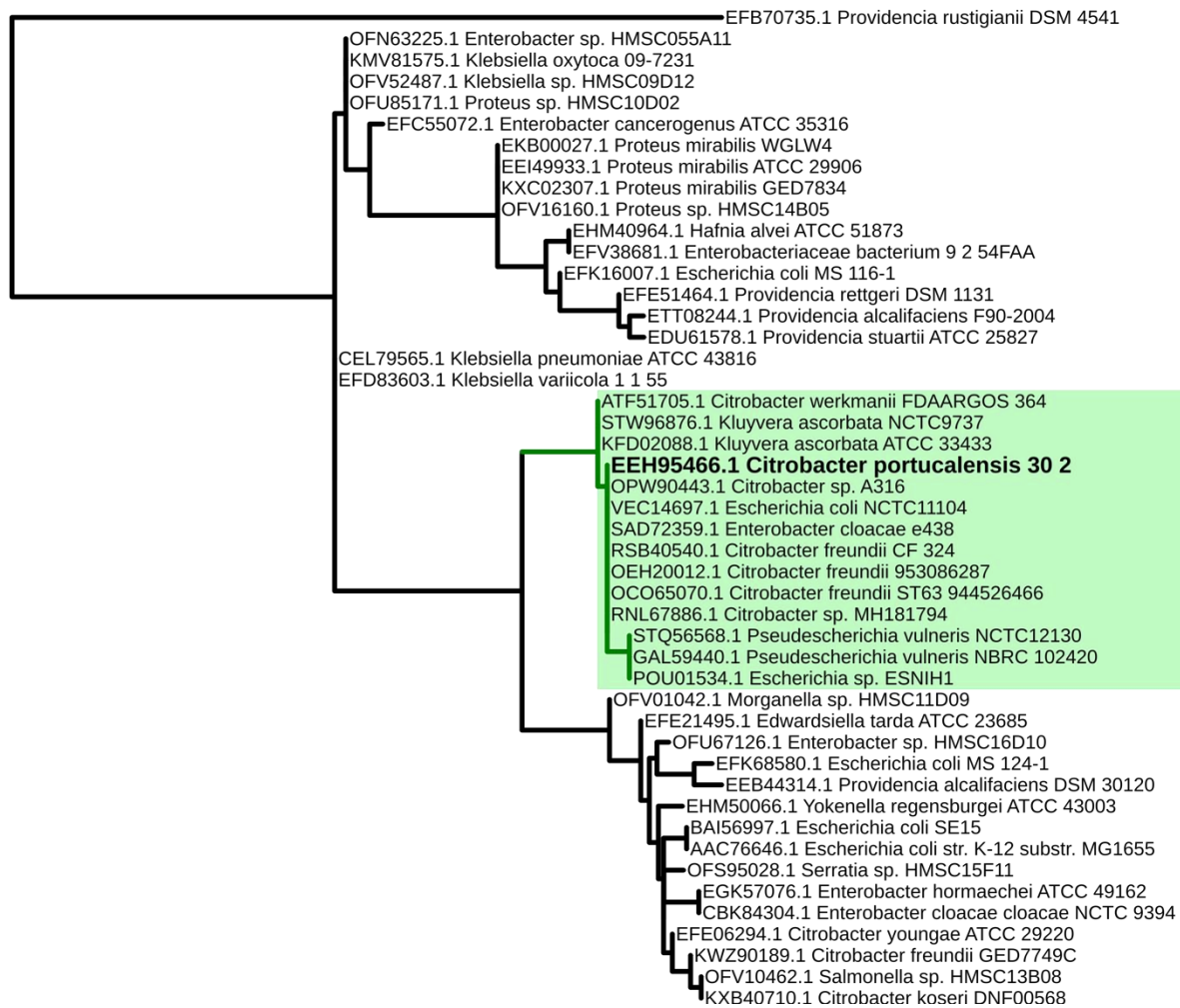

Supplement: FIG S4 [file mBio.02587-19-sf004.pdf]
