# Supplementary material for: Functional Multigenomic Screening of Human-Associated Bacteria for NF-κB-Inducing Bioactive Effectors
Source: mBio. 2019 Nov 19;10(6):e02587-19. doi: 10.1128/mBio.02587-19 (PMC6867899; doi:10.1128/mBio.02587-19)
Supplement: FIG S3 [file mBio.02587-19-sf003.pdf]

A

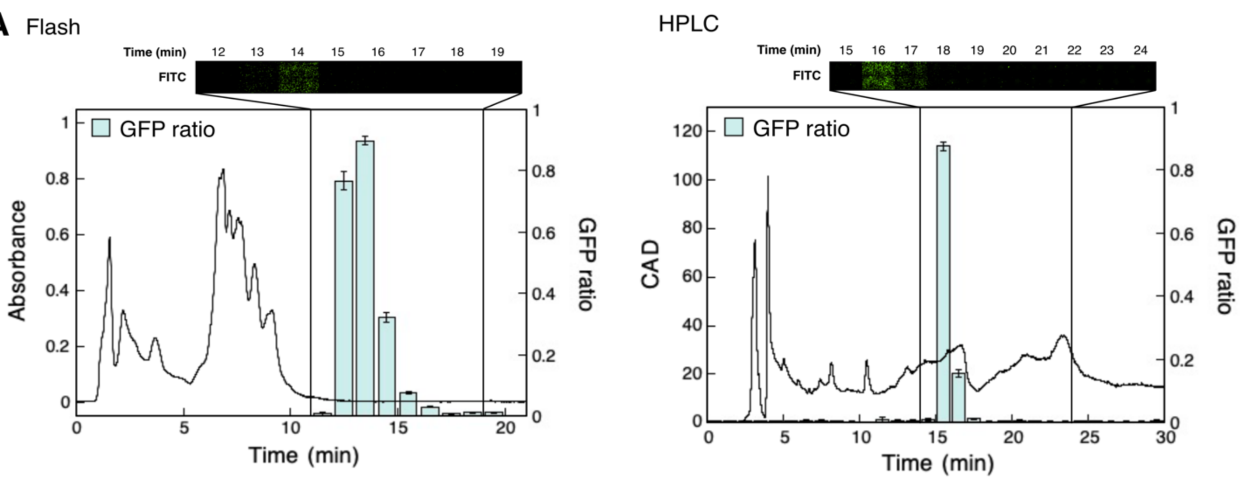

B

Biological Process	Term	Significant DEG in GOBP	Annotated Genes in GOBP	p-value	Adjusted p-value
GO:0002237	response to molecule of bacterial origin	11	284	0	0
GO:0006950	response to stress	28	3366	0	0
GO:0006952	defense response	20	1266	0	0
GO:0006954	inflammatory response	18	600	0	0
GO:0006955	immune response	19	1664	0	0
GO:0019221	cytokine-mediated signaling pathway	17	635	0	0
GO:0032496	response to lipopolysaccharide	11	273	0	0
GO:0034097	response to cytokine	18	996	0	0
GO:0034612	response to tumor necrosis factor	11	217	0	0
GO:0071216	cellular response to biotic stimulus	10	189	0	0
GO:0071219	cellular response to molecule of bacterial origin	10	166	0	0
GO:0071222	cellular response to lipopolysaccharide	10	160	0	0
GO:0071345	cellular response to cytokine stimulus	17	915	0	0
GO:0071356	cellular response to tumor necrosis factor	11	198	0	0
GO:0070098	chemokine-mediated signaling pathway	7	57	2.67E-11	2.82E-08
GO:0050900	leukocyte migration	11	353	5.31E-11	5.25E-08
GO:0051707	response to other organism	14	744	6.98E-11	5.81E-08
GO:0043207	response to external biotic stimulus	14	745	7.09E-11	5.81E-08
GO:1990868	response to chemokine	7	65	7.35E-11	5.81E-08
GO:1990869	cellular response to chemokine	7	65	7.35E-11	5.81E-08
GO:0009617	response to bacterium	12	475	8.72E-11	6.56E-08
GO:0009607	response to biotic stimulus	14	775	1.44E-10	1.04E-07
GO:0030593	neutrophil chemotaxis	7	72	1.85E-10	1.27E-07
GO:0051716	cellular response to stimulus	31	6291	2.26E-10	1.49E-07
GO:0048583	regulation of response to stimulus	25	3695	2.56E-10	1.62E-07
GO:1990266	neutrophil migration	7	81	4.12E-10	2.51E-07
GO:0023052	Signaling	29	5425	4.32E-10	2.53E-07
GO:0007154	cell communication	29	5465	5.56E-10	3.14E-07
GO:0007165	signal transduction	28	5015	5.9E-10	3.21E-07
GO:0071621	granulocyte chemotaxis	7	90	8.43E-10	4.44E-07
GO:0009966	regulation of signal transduction	22	2879	1.18E-09	6.03E-07
GO:0007166	cell surface receptor signaling pathway	21	2600	1.35E-09	6.68E-07
GO:0010469	regulation of signaling receptor activity	11	444	1.71E-09	8.2E-07
GO:0097530	granulocyte migration	7	102	1.94E-09	9.04E-07
GO:0006959	humoral immune response	8	160	2.09E-09	9.43E-07
