# Supplementary material for: Functional Multigenomic Screening of Human-Associated Bacteria for NF-κB-Inducing Bioactive Effectors
Source: mBio. 2019 Nov 19;10(6):e02587-19. doi: 10.1128/mBio.02587-19 (PMC6867899; doi:10.1128/mBio.02587-19)
Supplement: TABLE S4 [file mBio.02587-19-st004.pdf]

Accession	Organism	Isolation source	Mbeg 7 - d			Mbeg 7 - e			Mbeg 7 - f		
			Query Cover (%)	Expect Value	Identity (%)	Query Cover (%)	Expect Value	Identity (%)	Query Cover (%)	Expect Value	Identity (%)
**NZ_KI391984.1**	***Citrobacter portucalensis 30_2***	**Human (GI)**									
NZ_QRJT01000004.1	*Citrobacter portucalensis AM17-37*	Human (feces)	100	0.0	100	100	0.0	100	100	0.0	100
NZ_KI929266.1	*Citrobacter portucalensis UCI 32*	Human (urine)	100	0.0	99	100	0.0	99	100	0.0	100
NZ_RHWW01000036.1	*Citrobacter freundii CF_324*	Hospital sink	100	0.0	96	100	0.0	99	100	0.0	99
NZ_LJET02000242.1	*Citrobacter freundii 953086287*	Human	100	0.0	96	100	0.0	99	100	0.0	99
NZ_LJES02000001.1	*Citrobacter freundii ST63:944526466*	Human (catheter)	100	0.0	96	100	0.0	99	100	0.0	99
NZ_CP012554.1	*Citrobacter portucalensis P10159*	Human (urine)	100	0.0	96	100	0.0	99	100	0.0	99
NZ_JH414881.1	*Citrobacter portucalensis 4_7_47CFAA*	Human (GI)	100	0.0	95	100	0.0	99	100	0.0	99
NZ_LR134214.1	*Escherichia coli NCTC11104*	missing	100	0.0	95	100	0.0	99	100	0.0	99
NZ_RZIH01000004.1	*Citrobacter portucalensis CQ-CP1*	Amphibian	100	0.0	99	100	0.0	99	100	0.0	99
NZ_FKED01000006.1	*Enterobacter cloacae e438*	Human (blood)	100	0.0	95	100	0.0	99	100	0.0	99
NZ_RAPH01000007.1	*Citrobacter sp. MH181794*	missing	100	0.0	95	100	0.0	98	100	0.0	98
NZ_MVFZ01000023.1	*Citrobacter sp. A316*	water	100	0.0	96	100	0.0	98	100	0.0	98
NZ_CP023504.1	*Citrobacter werkmanii FDAARGOS_364*	Human (feces)	100	0.0	93	100	0.0	95	100	0.0	95
NZ_JMPL01000048.1	*Kluyvera ascorbata ATCC 33433*	Human (sputum)	100	0.0	87	100	0.0	91	100	0.0	93
NZ_UGNM01000001.1	*Kluyvera ascorbata NCTC9737*	Human (urine)	100	0.0	87	100	0.0	91	100	0.0	93
NZ_PQKR01000005.1	*Escherichia sp. ESNIH1*	waste water	-	-	-	-	-	-	34	1E-20	72
NZ_BBMZ01000018.1	*Pseudescherichia vulneris NBRC 102420*	Human (wound)	-	-	-	-	-	-	34	2E-12	71
NZ_UGGL01000002.1	*Pseudescherichia vulneris NCTC12130*	missing	-	-	-	-	-	-	36	2E-12	71
