# Supplementary material for: Functional Multigenomic Screening of Human-Associated Bacteria for NF-κB-Inducing Bioactive Effectors
Source: mBio. 2019 Nov 19;10(6):e02587-19. doi: 10.1128/mBio.02587-19 (PMC6867899; doi:10.1128/mBio.02587-19)
Supplement: TABLE S5 [file mBio.02587-19-st005.pdf]

| | | |
|---|---|---|
| | | **Library cosmids sequencing primers (pJWC1 cloning site)** |
| | Forward | GGCAACTACAGCTCAGGCGAC |
| | Reverse | CCTCGCCTTGGTAGCCATC |
| **Mbeg** | | **Sub-cloning PCR amplification primers** |
| 1 | Forward | *GAGA* **TCTAGA** CTTTAA GAAGGAG ATATACC ATGAAGTTGAAAACTTTAGTGATTG |
| | Reverse | *GAGA* **CTCGAG** TCATTGTTTGATTCGTCCTG |
| 2 | Forward | *GAGA* **TCTAGA** CTTTAA GAAGGAG ATATACC ATGAAAAATAAAAAAACGATACGTT |
| | Reverse | *GAGA* **CTCGAG** CTATTTCTTTAAAGGCAGATCAAGC |
| 3 | Forward | *GAGA* **TCTAGA** CTTTAA GAAGGAG ATATACC ATGGGGAAGCTGAGAATATT |
| | Reverse | *GAGA* **CTCGAG** CTACCTTGTAGTGTATGTCAAG |
| 4 | Forward | *GAGA* **TCTAGA** CTTTAA GAAGGAG ATATACC ATGTTGAAAGCAGAATGTCTTAC |
| | Reverse | *GAGA* **CTCGAG** TTAAGGCAGCGAATTATAATATTCG |
| 5 | Forward | *GAGA* **TCTAGA** CTTTAA GAAGGAG ATATACC ATGTTGTCTATTTTCCCACCA |
| | Reverse | *GAGA* **CTCGAG** TCATGCTTTATCCCCCG |
| 6 | Forward | *GAGA* **CCATGG** CGTATAGTGATGAAACAAATAT |
| | Reverse | *GAGA* **CTCGAG** TTATGCTTTACTTCCAAAACATTGC |
| 7 a-f | Forward | *GAGA* **TCTAGA** CTTTAA GAAGGAG ATATACC ATGCGTTTTCATGGCGATATG |
| | Reverse | *GAGA* **CTCGAG** TTACTGCAGGAATGGTTTTACATG |
| 7 a-c | Forward | *GAGA* **TCTAGA** CTTTAA GAAGGAG ATATACC ATGCGTTTTCATGGCGATATG |
| | Reverse | *GAGA* **CTCGAG** TTACAATGATTTGCGAATTGTTCTT |
| 7 d-f | Forward | *GAGA* **TCTAGA** CTTTAA GAAGGAG ATATACC ATGAATTCAAATACACAAACGCAAG |
| | Reverse | *GAGA* **CTCGAG** TTACTGCAGGAATGGTTTTACATG |
| 7 d | Forward | *GAGA* **TCTAGA** CTTTAA GAAGGAG ATATACC ATGAATTCAAATACACAAACGCAAG |
| | Reverse | *GAGA* **CTCGAG** TCATTTTTTAGCCCGTGATATTTTAT |
| 7 e | Forward | *GAGA* **TCTAGA** CTTTAA GAAGGAG ATATACC ATGAAAGTTTTAATTATTCGTAGAG |
| | Reverse | *GAGA* **CTCGAG** TTATGCTTCATCCATTGCG |
| 7 f | Forward | *GAGA* **TCTAGA** CTTTAA GAAGGAG ATATACC ATGATTAGAGCATTGAGAAAAAAAA |
| | Reverse | *GAGA* **CTCGAG** TTACTGCAGGAATGGTTTTACATG |
| | | **Sub-cloned cosmids sequencing primers (pET28c cloning site)** |
| | T7 | TAATACGACTCACTATAGGG |
| | T7-term | GCTAGTTATTGCTCAGCGG |
