# Supplementary material for: Functional Multigenomic Screening of Human-Associated Bacteria for NF-κB-Inducing Bioactive Effectors
Source: mBio. 2019 Nov 19;10(6):e02587-19. doi: 10.1128/mBio.02587-19 (PMC6867899; doi:10.1128/mBio.02587-19)
Supplement: TABLE S6 [file mBio.02587-19-st006.pdf]

Group*	Nucleotide Accession	Organism	Ligase	Heptosyltransferases				
Mbeg7	**KI391984**	***Citrobacter portucalensis strain 30_2***	EEH95466.1	EEH95463.1	EEH95462.2	EEH95469.2	EEH95464.2	EEH95465.1
Strains harboring Mbeg 7 homologs (NCBI GenBank)								
1	CP012554	*Citrobacter portucalensis P10159*						
1	JH414881	*Citrobacter portucalensis 4_7_47CFAA*						
1	KI929266	*Citrobacter portucalensis UCI 32*						
1	QRJT01000004	*Citrobacter portucalensis AM17-37*						
1	RZIH01000004	*Citrobacter portucalensis CQ-CP1*						
2	**CP023504**	***Citrobacter werkmanii FDAARGOS_364***	ATF51705.1	ATF51708.1	ATF51709.1	ATF51702.1	ATF51707.1	ATF51706.1
3	**LJES02000001**	***Citrobacter freundii ST63:944526466***	OCO65070.1	OCO64908.1	OCO64907.1	OCO64913.1	OCO64909.1	OCO64910.1
3	**LJET02000242**	***Citrobacter freundii 953086287***	OEH20012.1	OEH20007.1	OEH20006.1	OEH23152.1	OEH20008.1	OEH20009.1
3	**RHWW01000036**	***Citrobacter freundii CF_324***	RSB40540.1	RSB40537.1	RSB40536.1	RSB40543.1	RSB40538.1	RSB40539.1
4	**MVFZ01000023**	***Citrobacter sp. A316***	OPW90443.1	OPW90421.1	OPW90420.1	OPW90426.1	OPW90422.1	OPW90423.1
4	**RAPH01000007**	***Citrobacter sp. MH181794***	RNL67886.1	RNL67883.1	RNL67882.1	RNL67889.1	RNL67884.1	RNL67885.1
5	**LR134214**	***Escherichia coli NCTC11104***	VEC14697.1	VEC14703.1	VEC14705.1	VEC14691.1	VEC14701.1	VEC14699.1
6	**FKED01000006**	***Enterobacter cloacae e438***	SAD72359.1	SAD72274.1	SAD72245.1	SAD72441.1	SAD72305.1	SAD72336.1
7	**JMPL01000048**	***Kluyvera ascorbata ATCC 33433***	KFD02088.1	KFD02091.1	KFD02092.1	KFD02085.1	KFD02090.1	KFD02089.1
7	**UGNM01000001**	***Kluyvera ascorbata NCTC9737***	STW96876.1	STW96879.1	STW96880.1	STW96873.1	STW96878.1	STW96877.1
8	**PQKR01000005**	***Escherichia sp. ESNIH1***	POU01534.1	POU01530.1	POU01529.1	POU01537.1	POU01532.1	POU01533.1
9	**UGGL01000002**	***Pseudescherichia vulneris NCTC12130***	STQ56568.1	STQ56581.1	STQ56583.1	STQ56552.1	STQ56576.1	STQ56572.1
9	**BBMZ01000018**	***Pseudescherichia vulneris NBRC 102420***	GAL59440.1	GAL59436.1	GAL59435.1	GAL59443.1	GAL59438.1	GAL59439.1
Human microbiome Enterobacteriaceae (HMP catalog)								
10	**KQ959556**	***C. koseri DNF00568***	KXB40710.1	KXB40711.1	KXB40712.1	KXB40708.1		
10	**KQ956024**	***C.freundii GED7749C***	KWZ90189.1	KWZ90190.1	KWZ90191.1	KWZ90187.1		
10	**GG730302**	***C.youngae ATCC 29220***	EFE06294.1	EFE06293.1	EFE06292.1	EFE06296.1		
10	KQ956075	*C. koseri GED7778C*						
10	KQ956115	*C. koseri PSS_7778B*						
11	**GG739635**	***Edwardsiella tarda ATCC 23685***	EFE21495.1	EFE21497.1	EFE21498.1	EFE21491.1		
12	**GG704864**	***Enterobacter cancerogenus ATCC 35316***	EFC55072.1	EFC55075.1	EFC55076.1	EFC55070.1	EFC55074.1	
12	**FP929040**	***Enterobacter cloacae subsp. cloacae***	CBK84304.1	CBK84302.1	CBK84301.1	CBK84305.1		
12	**GL892088**	***Enterobacter hormaechei ATCC 49***	EGK57076.1	EGK57073.1	EGK57072.1	EGK57074.1	EGK57078.1	
13	**KV795449**	***Enterobacter sp. HMSC055A11***	OFU67126.1	OFU67133.1	OFU67131.1	OFU67132.1		
14	**KV787893**	***Enterobacter sp. HMSC16D10***	OFN63225.1	OFN63228.1	OFN63222.1	OFN63223.1	OFN63226.1	
15	**ADCU02000001**	***Enterobacteriaceae bacterium 9_2***	EFV38681.1	EFV38682.1	EFV38683.1	EFV38680.2	EFV38674.2	
16	**ADWT01000017**	***Escherichia coli 124-1***	EFK68580.1	EFK68579.1	EFK68578.1	EFK68588.1		
16	JH594565	*Escherichia coli*						

		4_1_47FAA					
16	NZ_GG669101	*Escherichia coli 83972*					
16	GG657390	*Escherichia coli D9*					
16	KQ961129	*Escherichia coli JPH264*					
16	KQ961245	*Escherichia coli JPH493*					
16	GG773041	*Escherichia coli MS 110-3*					
16	GG771839	*Escherichia coli MS 115-1*					
16	GG772880	*Escherichia coli MS 117-3*					
16	ADWU01000013	*Escherichia coli MS 119-7*					
16	GG773151	*Escherichia coli MS 153-1*					
16	GG773555	*Escherichia coli MS 16-3*					
16	GG771932	*Escherichia coli MS 182-1*					
16	GG774232	*Escherichia coli MS 185-1*					
16	GG772483	*Escherichia coli MS 187-1*					
16	GG771441	*Escherichia coli MS 198 -1*					
16	GG772742	*Escherichia coli MS 21-1*					
16	GG772263	*Escherichia coli MS 45-1*					
16	GG774449	*Escherichia coli MS 57-2*					
16	GG774366	*Escherichia coli MS 60-1*					
16	GG772360	*Escherichia coli MS 69-1*					
16	GG773240	*Escherichia coli MS 78-1*					
16	ADWR01000002	*Escherichia coli MS 79-10*					
16	GG771698	*Escherichia coli MS 84-1*					
16	ADWQ01000022	*Escherichia coli MS 85-1*					
16	ADWV01000001	*Escherichia coli MS-107-1*					
16	AP009240	*Escherichia coli SE11*					
16	JH815280	*Escherichia sp. 1_1_43*					
16	KQ235745	*Escherichia sp. 3_2_53FAA*					
17	**GG773408**	***Escherichia coli MS 116-1***	EFK16007.1	EFK16008.1	EFK16009.1	EFK16026.1	
17	GG772078	*Escherichia coli MS 146-1*					
17	GG773607	*Escherichia coli MS 175-1*					
17	GG774093	*Escherichia coli MS 196-1*					
17	JH660545	*Escherichia sp. 4_1_40B*					
18	**AP009378**	***Escherichia coli SE15***	BAI56997.1	BAI56995.1	BAI56996.1	BAI56998.1	BAI57007.1
18	**U00096.3**	***Escherichia coli str. K-12 substr. MG1655***	AAC76646.1	AAC76644.1	AAC76645.1	AAC76647.1	AAC76656.1
19	**JH417535**	***Hafnia alvei ATCC 51873***	EHM40964.1	EHM40965.1	EHM40966.1	EHM40957.1	EHM40963.1
19	KV824216	*Hafnia sp. HMSC23F03*					
20	**KQ235800**	***Klebsiella oxytoca 09-7231***	KMV81575.1	KMV81578.1	KMV81573.1	KMV81572.1	KMV81576.1
20	**CDMU01000001**	***Klebsiella pneumoniae ATCC 43816***	CEL79565.1	CEL79567.1	CEL79568.1	CEL79564.1	CEL79562.1
20	**KV793429**	***Klebsiella sp. HMSC09D12***	OFV52487.1	OFV52484.1	OFV52489.1	OFV52490.1	OFV52486.1
20	**GG745511**	***Klebsiella sp. 1_1_55***	EFD83603.1	EFD83605.1	EFD83606.1	EFD83600.1	EFD83602.1
20	JH603143	*Klebsiella oxytoca 10-5243*					
20	KQ235774	*Klebsiella oxytoca 10-5244*					
20	JH603137	*Klebsiella oxytoca 10-5245*					
20	JH603146	*Klebsiella oxytoca 10-5246*					
20	KQ235785	*Klebsiella oxytoca 10-5248*					
20	KQ235791	*Klebsiella oxytoca 10-5249*					
20	JAKX01000067	*Klebsiella oxytoca KA-2*					
20	JAKY01000070	*Klebsiella oxytoca OK-1*					
20	KQ956899	*Klebsiella pneumoniae MJR8396D*					
20	KV792996	*Klebsiella sp. HMSC16C06*					
20	KV789631	*Klebsiella sp. HMSC22F09*					
20	ALNJ01000043	*Klebsiella sp. OBRC7*					
20	GG703527	*K. pneumoniae ATCC 13884*					
20	JH930438	*K. pneumoniae WGLW1*					
20	JH930419	*K. pneumoniae WGLW2*					
20	JH930422	*K. pneumoniae WGLW3*					
20	JH930429	*K. pneumoniae WGLW5*					
20	JH414801	*Klebsiella sp. 4_1_44FAA*					
20	KV792621	*Klebsiella sp. HMSC16A12*					

20	KV789928	*Klebsiella sp. HMSC25G12*							
20	GL882715	*Klebsiella sp. MS 92-3*							
21	**KV791672**	***Morganella sp. HMSC11D09***	OFV01042.1	OFV01045.1	OFV01049.1	OFV01050.1			
22	**GG668582**	***Proteus mirabilis ATCC 29906***	EEI49933.1	EEI49920.1	EEI49919.1	EEI49927.1	EEI49921.1	EEI49924.1	EEI49936.1
23	**KQ960958**	***Proteus mirabilis GED7834***	KXC02307.1	KXC02297.1	KXC02296.1	KXC02300.1	KXC02298.1	KXC02310.1	
23	GG661996	*Proteus penneri ATCC 35198*							
23	JH815508	*Proteus mirabilis WGLW6*							
24	**JH815533**	***Proteus mirabilis WGLW4***	EKB00027.1	EKB00022.1	EKB00016.1	EKB00015.1	EKB00018.1	EKB00030.1	
25	**KV791997**	***Proteus sp. HMSC14B05***	OFU85171.1	OFU85168.1	OFU85173.1	OFU85174.1	OFU85170.1		
26	**KV791209**	***Proteus sp. HMSC10D02***	OFV16160.1	OFV16167.1	OFV16169.1	OFV16170.1	OFV16157.1		
27	**ABXW01000070**	***Providencia alcalifaciens DSM 30120***	EEB44314.1	EEB44309.1	EEB44308.1	EEB44322.1			
28	**JACS01000012**	***Providencia alcalifaciens F90-2***	ETT08244.1	ETT08271.1	ETT08276.1	ETT08282.1			
28	**GG705271**	***Providencia rettgeri DSM 1131***	EFE51464.1	EFE51471.1	EFE51472.1	EFE51460.1			
28	**DS607671**	***Providencia stuartii ATCC 25827***	EFB70735.1	EFB70719.1	EFB70720.1	EFB70708.1			
28	JALA01000018	*Providencia alcalifaciens PAL-1*							
28	JAKZ01000054	*Providencia alcalifaciens PAL-2*							
28	AZYZ01000023	*Providencia alcalifaciens PAL-3*							
28	JALC01000028	*Providencia alcalifaciens R90-1*							
28	JALB01000006	*Providencia alcalifaciens RIMD*							
29	**GG703822**	***Providencia rustigianii DSM 4541***	EDU61578.1	EDU61586.1	EDU61587.1	EDU61574.1			
30	**KV791808**	***Salmonella sp. HMSC13B08***	OFV10462.1	OFV10446.1	OFV10444.1	OFV10447.1			
31	**KV792405**	***Serratia sp. HMSC15F11***	OFS95028.1	OFS95032.1	OFS95026.1	OFS95025.1	OFS95035.1	OFS95030.1	
31	GG753567	*Serratia odorifera DSM 4582*							
32	**JH417870**	***Yokenella regensburgei ATCC 43003***	EHM50066.1	EHM50064.1	EHM50063.1	EHM50069.1	EHM50073.1		
